# Discovery of Quinazoline-2,4(1*H*,3*H*)-Dione Derivatives as Potential Antibacterial Agent: Design, Synthesis, and Their Antibacterial Activity

**DOI:** 10.3390/molecules27123853

**Published:** 2022-06-15

**Authors:** Nader M. Boshta, Farag A. El-Essawy, Mohammed B. Alshammari, Safaa G. Noreldein, Osama M. Darwesh

**Affiliations:** 1 Chemistry Department, Faculty of Science, Menoufia University, Shebin El-Koam 32511, Egypt; chemsafaagoda@gmail.com; 2 Preparatory Year Deanship, Basic Science Department, Prince Sattam Bin Abdulaziz University, P.O. Box 151, Alkharj 11942, Saudi Arabia; f.elessawy@psau.edu.sa; 3 College of Sciences and Humanities, Prince Sattam Bin Abdulaziz University, P.O. Box 151, Alkharj 11942, Saudi Arabia; m.alshammari@psau.edu.sa; 4 Environmental Microbiology and Nanotechnology Group, Agricultural Microbiology Department, National Research Centre, Cairo 12622, Egypt; darweshosama@yahoo.com

**Keywords:** synthesis, quinazoline, quinazoline-2,4(1*H*,3*H*)-dione, antimicrobial, fluoroquinolones

## Abstract

In this paper, we report on the design and synthesis of a novel series of quinazoline-2,4(1*H*,3*H*)-dione derivatives as fluoroquinolone-like inhibitors of bacterial gyrase and DNA topoisomerase IV to identify and develop antimicrobial agents to prevent bacterial resistance problems. Their structures were confirmed using spectroscopic analyses (IR, NMR, and EI-MS). The novel quinazoline-2,4(1*H*,3*H*)-dione derivatives were evaluated for their antimicrobial activities against Gram-positive and Gram-negative bacterial strains using the Agar well diffusion method to study the antimicrobial activities and compared them with the standard drugs. Most compounds displayed moderate activity. Among the tested compounds, the most promising compounds **13** and **15** provided broad bioactive spectrum against Gram-positive and Gram-negative strains compared to the standard drugs.

## 1. Introduction

Nitrogen heterocycles are among the most notable scaffolds for pharmaceuticals, in which quinazoline is one of those scaffolds that exists in a total of nine U.S. FDA approved pharmaceuticals. Several reports have revealed that the unique quinazoline scaffolds possess several and diverse pharmacological activities such as antimicrobial, antimalarial, anticonvulsant, anti-inflammatory, antihypertensive, cholinesterase inhibitory, anti-diabetic, and anticancer activities [1]. Among the most important quinazoline families are quinazoline-2,4(1*H*,3*H*)-dione derivatives, and the various studies have reported several analogues representing a wide variety of biological activities, for example, antimicrobial [2,3,4,5,6,7,8,9,10,11,12,13,14,15,16,17,18], anticancer [19,20,21,22,23,24,25,26,27,28,29,30,31,32], antiplatelet [33], antihypertensive [34], antioxidant [35], anticonvulsant [36,37,38], anti-inflammatory [39], phosphodiesterase (PDE) 4 inhibition [40], 5-HT3A receptor antagonist [41], and cyclin-dependent kinase 5 (CDK5) inhibition [42].

**Figure 1:**
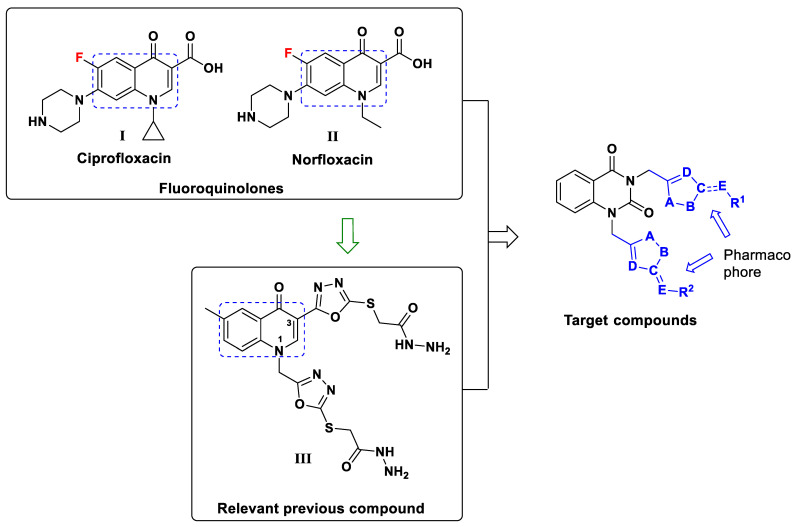
The design of the target compounds based on the relevant previous compound [48].

Fluoroquinolones, for example, I and II, are fatal and decisive antibacterial agents to exceedingly control many bacterial diseases [43]. However, many bacterial strains are resistant to such fluoroquinolone-like compounds [44,45,46]. Therefore, one way to avoid the resistance problems is to identify new derivatives to address the resistant mutant. On the other hand, a comprehensive study proved that quinazoline-2,4(1*H*,3*H*)-dione derivatives are fluoroquinolone-like inhibitors of bacterial gyrase and DNA topoisomerase IV [5,47]. In a continuation of our previous work on the synthesis and structural-activity relationships (SAR) of a novel series of 1,3-disubistituted quinoline derivatives [48] due to the current need to identify and develop inhibitors to prevent the bacterial resistance. Therefore, we selected the quinazoline-2,4(1*H*,3*H*)-dione scaffold to study and check the substituents’ effect at the same 1- and 3-positions on the quinazoline-dione ring (Figure 1). The most active compound discovered in our previous work was compound III, as shown in Figure 1.

## 2. Results and Discussion

### 2.1. Chemistry

**Scheme 1:**
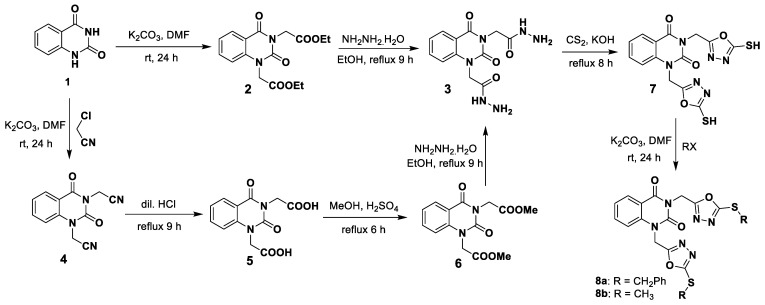
The synthesis of the target compounds **3**–**8**.

The structural–activity relationship study of the previous 1,3-disubistituted quinoline derivatives suggested that the antibacterial activity could be increased by performing synthetic modification at the 1- and 3-positions, interestingly, the incorporation of the oxadiazole rings at both positions led to a significantly more potent compound (compd. III, Figure 1). Therefore, one part of this study aimed to investigate the incorporation of the same heterocyclic ring at the 1- and 3-positions of the quinazoline-2,4(1*H*,3*H*)-dione scaffold, as depicted in Scheme 1. Appropriately, quinazoline-2,4(1*H*,3*H*)-dione (**1**) was either commercially available or synthesized from anthranilic acid [49], followed by *N*-alkylation with ethyl chloroacetate in stirring dimethyl formamide in the presence of potassium carbonate at room temperature. The key intermediate 2,2′-(2,4-dioxoquinazoline-1,3(2*H*,4*H*)-diyl)di(acetohydrazide) (**3**) was afforded via the hydrazinolysis reaction of **2** at the reflux condition. Alternatively, synthesized from 2,2′-(2,4-dioxoquinazoline-1,3(2*H*,4*H*)-diyl)diacetonitrile (**4**), the hydrolysis of the respective nitrile groups to afford the acid derivative **5**, was followed by esterification in the presence of sulfuric acid and methanol. The resulting dimethyl 2,2′-(2,4-dioxoquinazoline-1,3(2*H*,4*H*)-diyl)diacetate (**6**) underwent the hydrazinolysis reaction to afford the key intermediate **3** in a good yield of 72%. The crucial derivative **7** was prepared from dihydrazide **3** by treatment with carbon disulfide in the presence of potassium hydroxide. Finally, the target compounds **8a** and **8b** were synthesized via the alkylation reaction of compound **7** with benzyl chloride or methyl iodide, respectively. The structure of the synthesized derivatives was confirmed using mass spectrometry and NMR. The structure of the key intermediate **3** was obviously confirmed by the ^1^H-NMR spectrum, which showed the demise of the triplet signals at 1.21 ppm as well as the multiple signal at 4.17 ppm arising from the two COOEt groups of compound **2**. Moreover, the appearance of signals assignable to the new (CONHNH_2_) groups at 9.30, 9.21, and 4.27 ppm. The cyclization of compound **3** resulted into compound **7** which was confirmed by the absence of the last-mentioned signals for (NH & NH_2_) groups in the ^1^H-NMR spectrum. Compounds **8a** and **8b** were confirmed by the appearance of new signals at 4.44 ppm arising from the new methylene fragments in the ^1^H-NMR spectrum for **8a** and in parallel with the appearance of a new signal at 2.67 ppm arising from the new methyl fragments for **8b**.

**Scheme 2:**
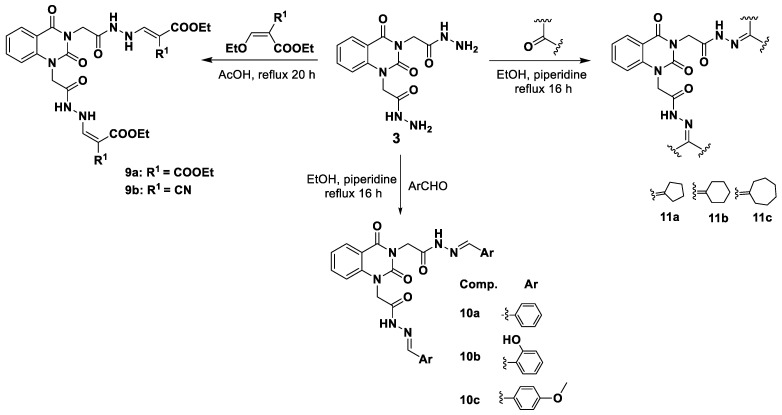
The synthesis of the target compounds **9**–**11**.

The key intermediate **3** could provide the opportunity for further novel quinazoline-2,4(1*H*,3*H*)-dione derivatives and have been prepared based on the synthetic producers displayed in Scheme 2. The reaction of compound **3** with diethyl ethoxymethylenemalonate or ethyl ethoxymethylene cyanoacetate under the reflux condition in acetic acid affording the malonate derivative **9a** or cyanoacetate derivative **9b** as a mixture of *E/Z* isomers, respectively. Furthermore, the direct condensation of diacetohydrazide **3** with aromatic aldehyde benzaldehyde, 2-hydroxybenzaldehyde, or 4-methoxybenzaldehyde to afford the corresponding arylidene **10a**, **10b**, or **10c**, respectively, as mixtures of *E/Z* isomers. Comparably, the condensation reaction with cyclic ketone cyclopentanone, cyclohexanone, or cycloheptanone afforded the corresponding cycloalkylidene derivatives **11a**, **11b**, or **11c**, respectively. The structure of the novel products **9a** and **9b** were confirmed by the demise of the two –NH_2_ groups in compound **3** and the appearance of new signals corresponding to the new methine protons (HN–*CH*=) at 7.69 and 8.20–8.21 ppm, respectively, in the ^1^H-NMR spectrum, in addition to the appearance of the characteristic signals of the new COOEt groups. Compounds **10a**, **10b**, and **10c** were confirmed by the ^1^H-NMR spectrum, in which the signals matching to the two amino groups in compound **3** were replaced by singlet signals assignable to the new Schiff base protons (N=CH–Ar) in the region of 8.12–8.44 ppm, in addition to the new aromatic protons because of the insertion of the aryl group of the appropriate aldehydes in the range of 6.87–8.36 ppm. Compounds **11a**, **11b**, and **11c** showed signals attributable to the new aliphatic protons as a result of the installation of cyclic aliphatic moieties of the appropriate cyclic ketones at the range 1.51–2.68 ppm.

**Scheme 3:**
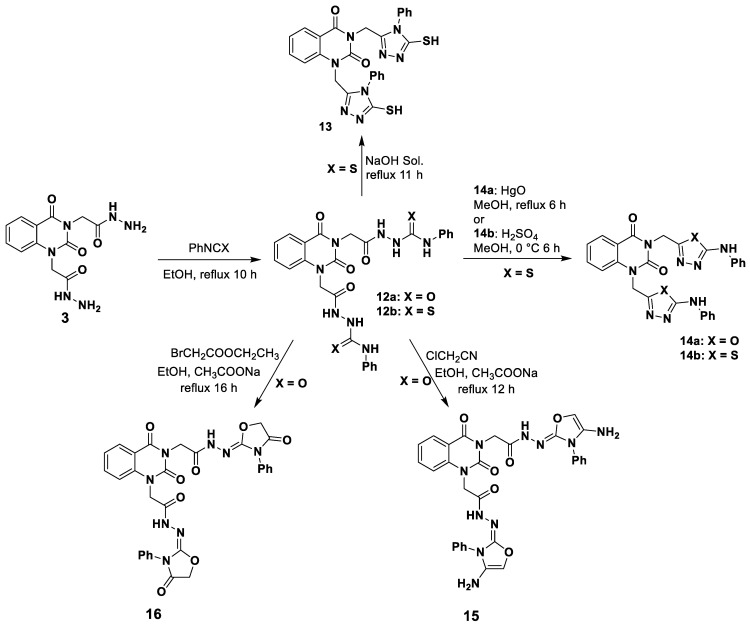
The synthesis of the target compounds **12**–**16**.

The reaction of diacetohydrazide **3** with phenyl isocyanate or phenyl isothiocyanate in ethanol under the reflux condition affords the corresponding disemicarbazide derivative **12a** or dithiosemicarbazide derivative **12b** in a good yield. Consequently, such derivatives could be practical as a template for further modifications to synthesize more substituted analogues at the 1- and 3-positions of the quinazoline-2,4(1*H*,3*H*)-dione scaffold, particularly with hetero cyclic moieties. The triazole, oxadiazole, or thiadiazole derivatives can be afforded during the cyclization reaction of **12b** based on the reaction medium. The cyclization of the last product **12b** in the presence of NaOH under reflux conditions afforded 1,3-bis((5-mercapto-4-phenyl-4*H*-1,2,4-triazol-3-yl)methyl)quinazoline-2,4(1*H*,3*H*)-dione (**13**). Otherwise, the cyclization of the dithiosemicarbazide derivative **12b** in the presence of mercuric oxide HgO under reflux conditions gave 1,3-bis((5-(phenylamino)-1,3,4-oxadiazol-2-yl)methyl)quinazoline-2,4(1*H*,3*H*)-dione **14a** while the cyclization via stirring at 0 °C in the presence of sulfuric acid provided 1,3-bis((5-mercapto-4-phenyl-4*H*-1,2,4-triazol-3-yl)methyl)quinazoline-2,4(1*H*,3*H*)-dione **14b**. On the other hand, disemicarbazide derivative **12a** was easily intermolecular-cyclized, either with 2-chloroacetonitrile or ethyl bromoacetate in the presence of anhydrous sodium acetate in absolute ethanol, and provided compounds **15** and **16**, respectively, in a very good yield (Scheme 3). Compounds **12a** and **12b** were confirmed by ^1^H-NMR, in which the signals corresponding to the (NH_2_) protons in compound **3** were replaced by signals assignable to the new (CO*NH*Ph or CS*NH*Ph) protons at the range of 9.30–11.00 ppm and the new aromatic protons at the range of 7.19–8.10 ppm. The cyclization of compound **12b** afforded compound **13**, which was confirmed by the absence of signal (CS*NH*Ph) protons with the appearance of new typical signals of the (SH) proton at 13.79 ppm. Compounds **14a** and **14b** were confirmed by the absence of singlet signals at the range of 9.41–11.00 ppm, which was related to the (NHNHCSNH) protons and by the presence of new singlet signals at the range of 10.44–10.53 ppm related to (NHPh) in the ^1^H-NMR spectrum. Similarly, compounds **15** and **16** were confirmed by the absence of singlet signals at the range of 8.32–10.09 ppm, which was related to the (NHNHCONH) protons and by the presence of new signal at 4.25 ppm, which was related to the new (NH_2_) group for compound **15**, and new signals at 4.52 and 4.72 ppm, which were related to the new (–OCH_2_CO) groups for compound **16**.

### 2.2. Antibacterial Activity

The antimicrobial activities of the synthesized compounds were examined against some targeted pathogenic microorganisms obtained from the American Type of Culture Collection (ATCC; Rockville, MD, USA). The compounds were evaluated for antimicrobial activity against *Staphylococcus aurous* ATCC-47077 (St.), *Listeria monocytogenes* ATCC-35152 (List.), *Escherichia coli* ATCC-25922 (E.C.), *Salmonella typhi* ATCC-15566 (Salm.), and *Candida albicans* ATCC-10231 (C. Alb.) [50,51]. The stock cultures of pathogens used in this study were maintained on nutrient agar slants at 4°. The Agar well diffusion method was employed to study the antimicrobial activities according to the method described by Kheiralla et al. [52] and Abdelhameed et al. [53] in which the zone of inhibition for the tested compound was measured by a ruler to determine its size (in mm) and compared with that produced by the reference antibacterial drugs ampicillin and vancomycin. The minimum inhibitory concentration (MIC) calculation of the synthesized compounds was also performed according to a slightly modified previous method [54,55] in which the MIC was defined as the concentration at which the bacteria and yeast did not show visible growth with respect to the positive control.

The synthesized intermediate **3** and compound **9b** both showed no activity against most of the tested Gram-positive and Gram-negative strains, except for the *Candida albicans* strain, which is quite sensitive to those derivatives with an inhibition zone value of 11 mm and MIC value of 80 mg/mL, and so more effective than the reference antibacterial drug ampicillin. Compounds **13** and **15** gave broad bioactive spectrum against Gram-positive and Gram-negative strains in which compound **13** with triazole moieties at the 1- and 3-poisitions of the quinazoline-2,4-dione backbone showed moderate activity with a measured inhibition zone value of 9 mm against the *Staphylococcus aurous* strain when compared with the reference antibacterial drugs ampicillin and vancomycin. It also showed a specific activity against the *Escherichia coli* strain with inhibition zone values of 15 mm and MIC value of 65 mg/mL, which are equipotent to the reference drugs. Compound **13** significantly inhibited the growth of the *Candida albicans* strain with an inhibition zone value of 11 mm, and surpassed the efficacy of the reference antibacterial drug ampicillin. It was worthwhile to note that compound **15** showed moderate activities against *Staphylococcus aurous*, *Escherichia coli*, and *Candida albicans* strains with inhibition zone values in the range of 10–12 mm and MIC values of 80, 75, and 77 mg/mL, respectively. On the other hand, the synthesized quinazoline-2,4-dione derivatives **14a** and **14b** with oxadiazole and thiadiazol moieties at the 1- and 3-poisitions of the quinazoline backbone could effectively inhibit the growth of *Staphylococcus aurous* and *Candida albicans* strains in which both compounds exhibited moderate activity against *Staphylococcus aurous* with inhibition zone values of 12 and 13 mm and MIC values of 70 and 75 mg/mL, respectively. The *Candida albicans* strain was quite sensitive to compound **14a** with an inhibition zone value of 12 mm, which was more effective than the standard drug ampicillin, as depicted in Table 1.

In this context and concerning the structure–activity relationship (SAR), the synthesized compounds **13**, **14a**, **14b**, and **15** indicated that the integration of triazole (compound **13**), oxadiazole (compound **14a**), thiadiazole (compound **14b**), or oxazole (compound **15**) across different linkers at position *N*-1 and *N*-3 of the quinazoline nucleus was essential for antimicrobial activity. It is worthwhile mentioning that our previous work that included the synthesis and SAR of a novel series of 1,3-disubistituted quinoline derivatives [49] concluded the same results. Therefore, molecular hybridization via incorporating these moieties (triazole, oxadiazole, thiadiazole or oxazole) and others in the 1,3-positions of the quinoline or quinazoline nucleus might be thought to attain more potent antibacterial compounds.

## 3. Experimental

General Methods: All solvents and reagents were of analytical grade. The reactions were monitored by aluminum oxide thin layer chromatography TLC (silica gel 60F254, Merck, Darmstadt, Germany). Melting points were determined and were uncorrected on a melting point apparatus (Stuart Scientific, Stone, Staffordshire, UK). Mass spectra were measured on a GC MS-QP 1000 EX Mass Spectrometer (Shimadzu, Tokyo, Japan). NMR spectra were carried out with a 300 MHz at Cairo University with 400 MHz at Ulm University, Germany. Chemical shifts (δ) are reported in parts per million (ppm) relative to the respective solvent as the internal standard and the standard abbreviations were used. The required starting material **1** was prepared by adopting the earlier reported procedures [40]. Compounds **2**, **3**, **4**, and **5** were known before and explored in scientific research [56,57].

The spectral data for the target compounds are shown in the Electronic Supplementary Materials.

### 3.1. Diethyl 2,2′-(2,4-Dioxoquinazoline-1,3(2H,4H)-diyl)diacetate (***2***)

A mixture of **1** (1.0 g, 6.2 mmol) and potassium carbonate anhydrous (K_2_CO_3_) (1.7 g, 12.4 mmol) in dimethylformamide (DMF) (10 mL) was stirred for about 15 min at room temperature. Then, ethyl chloroacetate (1.5 g, 12.4 mmol) was added. The reaction mixture was stirred for 24 h at room temperature, poured onto ice water, and the solid product was collected by filtration, washed with water, and dried to give **2** (1.5 g, 73%) as a white powder; m.p. 118–120 °C; ^1^H-NMR (400 MHz, DMSO-d_6_) δ 1.21 (t, *J* = 7.07 Hz, 6H, 2(-CH_3_)), 4.17 (q, 4H, 2(-*CH_2_*-CH_3_)), 4.72 (s, 2H, -CH_2_-), 4.99 (s, 2H, -CH_2_-), 7.37 (t, *J* = 7.05 Hz, 1H, Ar-H), 7.45 (d, *J* = 8.21 Hz, 1H, Ar-H), 7.80 (t, *J* = 6.92 Hz, 1H, Ar-H), 8.11 (d, *J* = 8.66 Hz, 1H, Ar-H) ppm. M.S (EI, *m*/*z*, 70 eV): Calcd. = 334.33 and Found = 335.2 [M^+^ + 1].

### 3.2. 2,2′-(2,4-Dioxoquinazoline-1,3(2H,4H)-diyl)di(acetohydrazide) (***3***)

A mixture of **2** (1.0 g, 3.0 mmol) and hydrazine hydrate (0.3 g, 6.0 mmol) in a 25 mL one-necked flask was dissolved in (10 mL) absolute ethanol. The mixture was heated under reflux for 9 h, subsequently, the solvent was concentrated under high vacuum. The resulting precipitate was filtered off, washed with ethanol, and dried to give **3** (0.8 g, 88%) as a white powder; m.p. 262–264 °C; ^1^H-NMR (400 MHz, DMSO-d_6_) δ 4.27 (br.s, 4H, 2(-NH_2_)), 4.52 (s, 2H, -CH_2_-), 4.74 (s, 2H, -CH_2_-), 7.24 (d, *J* = 8.76 Hz, 1H, Ar-H), 7.33 (t, *J* = 6.98 Hz, 1H, Ar-H), 7.76 (t, *J* = 7.01 Hz, 1H, Ar-H), 8.06 (d, *J* = 8.59 Hz, 1H, Ar-H), 9.21 (s, 1H, -NH-), 9.30 (s, 1H, -NH-) ppm. ^13^C-NMR (100 MHz, DMSO-d_6_) δ 42.62, 45.03, 115.07, 122.99, 127.89, 135.29, 140.40, 150.45, 161.03, 165.88, 166.17, 169.87 ppm.

### 3.3. 2,2′-(2,4-Dioxoquinazoline-1,3(2H,4H)-diyl)diacetonitrile (***4***)

A mixture of **1** (1.0 g, 6.2 mmol) and potassium carbonate anhydrous (K_2_CO_3_) (1.7 g, 12.4 mmol) in DMF (10 mL) was stirred for about 15 min at room temperature. Then, chloroacetonitrile (0.94 g, 12.4 mmol) was added. The reaction mixture was stirred for 24 h at room temperature, poured onto ice water, where the solid product was collected by filtration, washed with water, and dried to give **4** (1 g, 68%) as an off white powder; m.p. 208–210 °C; ^1^H-NMR (500 MHz, DMSO-d_6_) δ 4.94 (s, 2H, -CH_2_-), 5.33 (s, 2H, -CH_2_-), 7.42 (t, *J* = 6.95 Hz, 1H, Ar-H), 7.63 (d, *J* = 8.68 Hz, 1H, Ar-H), 7.89 (t, *J* = 7.05 Hz, 1H, Ar-H), 8.12 (d, *J* = 8.22 Hz, 1H, Ar-H) ppm. M.S (EI, *m*/*z*, 70 eV): Calcd. = 240.22, Found = 240.05, 240.95 [M^+^], 241.95 [M^+^ + 1], 242.95 [M^+^ + 2].

### 3.4. 2,2′-(2,4-Dioxoquinazoline-1,3(2H,4H)-diyl)diacetic acid (***5***)

A mixture of **4** (1.0 g, 4.2 mmol) and diluted hydrochloric acid (1:1) (10 mL) was refluxed for 9 h, then the reaction mixture was cooled. The solid form was filtered off, washed with water, and dried to give **5** (1.0 g, 87%) as a yellow powder; m.p. 226–228 °C; ^1^H-NMR (300 MHz, DMSO-d_6_) δ 4.62 (s, 2H, -CH_2_-), 4.89 (s, 2H, -CH_2_-), 7.34 (t, *J* = 8.76 Hz, 1H, Ar-H), 7.41 (d, *J* = 8.59 Hz, 1H, Ar-H), 7.78 (t, *J* = 6.97 Hz, 1H, Ar-H), 8.09 (d, *J* = 8.75 Hz, 1H, Ar-H) ppm.

### 3.5. Dimethyl 2,2′-(2,4-Dioxoquinazoline-1,3(2H,4H)-diyl)diacetate (***6***)

A mixture of **5** (1.0 g, 3.6 mmol) in methanol (10 mL) and the catalytic amount of conc. sulfuric acid were refluxed for 6 h. The reaction mixture was cooled to form a solid that was filtered off, washed with water, and dried to give **6** (0.8 g, 73%) as a white powder; m.p. 122–124 °C; ^1^H-NMR (300 MHz, DMSO-d_6_) δ 3.71 (s, 6H, 2(-CH_3_-), 4.73 (s, 2H, -CH_2_-), 5.01 (s, 2H, -CH_2_), 7.37 (t, *J* = 7.04 Hz, 1H, Ar-H), 7.46 (d, *J* = 8.66 Hz, 1H, Ar-H), 7.80 (t, *J* = 7.12 Hz, 1H, Ar-H), 8.11 (d, *J* = 8.80 Hz, 1H, Ar-H) ppm.

### 3.6. 1,3-Bis((5-mercapto-1,3,4-oxadiazol-2-yl)methyl)quinazoline-2,4(1H,3H)-dione (***7***)

To a solution of potassium hydroxide (0.74 g, 13.2 mmol) in ethanol/water (7 mL/7 mL) were hydrazide **3** (2.0 g, 6.6 mmol) and carbon disulfide (1.0 g, 13.2 mmol) added. The reaction mixture was heated under reflux for 8 h, and subsequently, the solvent was concentrated under high vacuum before adding water and acidified with conc. hydrochloric acid. The formed solid was filtered off, washed with water, and the obtained compound was further purified by crystallizing from ethanol and dried to give **7** (1.9 g, 75%) as a white solid; m.p. 180–182 °C; ^1^H-NMR (500 MHz, DMSO-d_6_) δ 5.23 (s, 2H, -CH_2_-), 5.50 (s, 2H, -CH_2_-), 7.39 (t, *J* = 6.95 Hz, 1H, Ar-H), 7.64 (d, *J* = 8.59 Hz, 1H, Ar-H), 7.84 (t, *J* = 8.76 Hz, 1H, Ar-H), 8.11(d, *J* = 8.81 Hz, 1H, Ar-H) ppm. M.S (EI, *m*/*z*, 70 eV): Calcd. = 390.39, Found = 392.30 [M^+^ + 2].

### 3.7. General Procedure for the Synthesis of ***8a*** and ***8b***

A mixture of **7** (0.3 g, 0.8 mmol) and K_2_CO_3_ (0.22 g, 1.6 mmol) in DMF (3 mL) was stirred for about 30 min at room temperature. Then, benzyl chloride or methyl iodide (0.8 mmol) was added. The reaction mixture was stirred for 24 h at room temperature, poured onto ice water, and the solid product was collected by filtration and the obtained compounds were further purified by crystallizing from ethanol.

#### 3.7.1. 1,3-Bis((5-(benzylthio)-1,3,4-oxadiazol-2-yl)methyl)quinazoline-2,4(1*H*,3*H*)-dione (**8a**)

Yield (0.32 g, 74%) as a white solid; m.p. 126–128 °C; ^1^HNMR (500 MHz, DMSO-d_6_) δ 4.44 (s, 4H, 2(-CH_2_-)), 5.36 (s, 2H, (-SCH_2_-)), 5.63 (s, 2H, (-SCH_2_-)), 7.22 (t, *J* = 7.07 Hz, 1H, Ar-H), 7.31–7.36 (m, 10H, Ar-H), 7.60 (d, *J* = 9.88 Hz, 1H, Ar-H), 7.83 (t, *J* = 7.06 Hz, 1H, Ar-H), 8.13 (d, *J* = 8.99 Hz, 1H, Ar-H) ppm.

#### 3.7.2. 1,3-Bis((5-(methylthio)-1,3,4-oxadiazol-2-yl)methyl)quinazoline-2,4(1*H*,3*H*)-dione (**8b**)

Yield (0.3 g, 94%) as a white solid; m.p. 178–180 °C; ^1^H-NMR (400 MHz, DMSO-d_6_) δ 2.67 (s, 6H, 2(-CH_3_)), 5.37 (s, 2H, -CH_2_-), 5.63 (s, 2H, -CH_2_-), 7.39 (t, *J* = 7.04 Hz, 1H, Ar-H), 7.64 (d, *J* = 8.87 Hz, 1H, Ar-H), 7.83 (t, *J* = 7.08 Hz, 1H, Ar-H), 8.13 (d, *J* = 8.66 Hz, 1H, Ar-H) ppm. ^13^C-NMR (100 MHz, DMSO-d_6_) δ 14.42, 36.19, 38.65, 114.75, 115.11, 123.95, 128.39, 136.10, 139.44, 149.91, 160.60, 162.99, 163.33, 164.83, 165.39 ppm.

### 3.8. General Procedure for the Synthesis of ***9a*** and ***9b***

To a solution of **3** (0.2 g, 0.7 mmol) in absolute ethanol (2 mL), diethyl ethoxymethylenemalonate or ethyl ethoxymethylene cyanoacetate (1.4 mmol) was added and the reaction mixture was refluxed for 20 h. The reaction mixture was cooled to reach room temperature, poured into ice water, then the solid product was collected by filtration and washed with ethanol to give **9a** and **9b**, respectively.

#### 3.8.1. Tetraethyl 2,2′-(((2,2′-(2,4-Dioxoquinazoline-1,3(2*H*,4*H*)-diyl)bis(acetyl))bis(hydrazine-2,1-diyl))bis(methanylylidene))dimalonate (**9a**)

Yield (0.3 g, 71%) as a white solid; m.p. 128–130 °C; ^1^H-NMR (500 MHz, DMSO-d_6_) δ 1.16–1.21 (m, 12H, 4(-CH_3_)), 4.00 (q, 2H, -*CH_2_*CH_3_), 4.01–4.23 (m, 4H, 2(-*CH_2_*CH_3_)), 4.2 (q, 2H, -*CH_2_*CH_3_), 4.65 (s, 2H,-CH_2_-), 4.88 (s, 2H, -CH_2_-), 7.35 (t, *J* = 6.92 Hz, 1H, Ar-H), 7.69 (s, 2H, 2(-CH=)), 7.71 (d, *J* = 8.66 Hz, 1H, Ar-H), 7.8 (t, *J* = 7.08 Hz, 1H, Ar-H), 8.08 (d, *J* = 8.59 Hz, 1H, Ar-H), 10.06 (s, 1H, -NH-), 10.09 (s, 1H, -NH-), 11.04 (s, 1 H, -NH-), 11.06 (s, 1H, -NH-) ppm. M.S (EI, *m*/*z*, 70 eV): Calcd. = 646.61, Found = 648 [M^+^ + 2].

#### 3.8.2. Ethyl-2-cyano-3-(2-(2-(1-(2-(2-(-2-cyano-3-ethoxy-3-oxoprop-1-en-1-yl)hydrazinyl)-2-oxoethyl)-2,4-dioxo-1,4-dihydroquinazolin-3(2*H*)-yl)acetyl)hydrazinyl)acrylate (**9b**)

Yield (0.3 g, 83%) as a white solid; m.p. 158–160 °C; ^1^H-NMR (500 MHz, DMSO-d_6_) δ 1.26 (t, *J* = 6.95 Hz, 6H, 2(-CH_3_)), 4.22 (q, 4H, 2(-*CH_2_*CH_3_)), 5.38 (s, 2H, -CH_2_-), 5.64 (s, 2 H, -CH_2_-), 7.39 (t, *J* = 7.02 Hz, 1H, Ar-H), 7.65 (d, *J* = 8.66 Hz, 1H, Ar-H), 7.75 (t, *J* = 7.07 Hz, 1H, Ar-H), 7.89 (s, 1H, -CH=), 7.92 (s, 1H, -CH=), 8.1 (d, *J* = 8.77 Hz, 1H, Ar-H) ppm. M.S (EI, *m*/*z*, 70 eV): Calcd. = 552.50, Found = 553 [M^+^ + 1], 554 [M^+^ + 2].

### 3.9. General Procedure for the Synthesis of Compounds ***10a***, ***10b***, ***10c***, ***11a***, ***11b***, and ***11c***

A mixture of **3** (0.2 g, 0.7 mmol) and aldehyde or ketone (1.4 mmol) in absolute ethanol (10 mL) in the presence of five drops of piperidine, the reaction mixture was refluxed for 16 h. The formed solid was filtered off and dried. The obtained compounds were further purified by crystallizing from ethanol.

#### 3.9.1. 2,2′-(2,4-Dioxoquinazoline-1,3(2*H*,4*H*)-diyl)bis(*N*′-(-benzylidene)acetohydrazide) (**10a**)

Yield (0.25 g, 81%) as a white solid; m.p. 320–322 °C; ^1^H-NMR (500 MHz, DMSO-d_6_) δ 5.10 (s, 2H, -CH_2_-), 5.36 (s, 2H, -CH_2_-), 7.34–8.1 (m, 14H, Ar-H), 8.2 (s, 1H, -N=CH-),), 8.21 (s, 1H, -N=CH-), 11.77 (s, 1H, -NH-), 11.89 (br.s, 1H, -NH-) ppm. M.S (EI, *m*/*z*, 70 eV): Calcd. = 482.50, Found = 482.10 [M^+^].

#### 3.9.2. 2,2′-(2,4-Dioxoquinazoline-1,3(2*H*,4*H*)-diyl)bis(*N*′-(-2 hydroxybenzylidene)acetohydrazide) (**10b**)

Yield (0.29 g, 88%) as a white solid; m.p. 312–314 °C; ^1^H-NMR (500 MHz, DMSO-d_6_) δ 5.07 (s, 2H, -CH_2_-), 5.33 (s, 2H, -CH_2_-), 6.87–8.36 (m, 12H, Ar-H), 8.41 (s, 1H, -N=CH-), 8.44 (s, 1H, -N=CH-), 10.05 (br.s, 1H, -NH-), 10.95 (br.s, 1H, -NH-), 11.68 (br.s, 1H, -OH), 11.95 (br.s, 1H, -OH) ppm. M.S (EI, *m*/*z*, 70 eV): Calcd. = 514.50, Found = 514.10 [M^+^], 515.10 [M^+^ + 1], 516.10 [M^+^ + 2].

#### 3.9.3. 2,2′-(2,4-Dioxoquinazoline-1,3(2*H*,4*H*)-diyl)bis(*N*′-(-4-methoxybenzylidene)acetohydrazide) (**10c**)

Yield (0.3 g, 86%) as a yellow solid; m.p. 310–312 °C; ^1^H-NMR (500 MHz, DMSO-d_6_) δ 3.79 (s, 6H, 2(-CH_3_)), 5.08 (s, 2H, (-CH_2_-)), 5.33 (s, 2H, (-CH_2_-)), 6.98–8.1 (m, 12H, Ar-H), 8.12 (s, 1H, -N=CH-), 8.15 (s, 1H, -N=CH-), 11.60 (br.s, 1H, -NH-), 11.68 (br.s, 1H, -NH-) ppm. M.S (EI, *m*/*z*, 70 eV): Calcd. = 542.55, Found = 542.10 [M^+^], 543.10 [M^+^ + 1], 544.10 [M^+^ + 2].

#### 3.9.4. 2,2′-(2,4-Dioxoquinazoline-1,3(2*H*,4*H*)-diyl)bis(*N*′-cyclopentylideneacetohydrazide) (**11a**)

Yield (0.22 g, 79%) as a white solid; m.p. 236–238 °C; ^1^H-NMR (500 MHz, DMSO-d_6_) δ 1.67–1.71 (m, 4H, 2(-CH_2_-)), 1.75–1.78 (m, 4H, 2(-CH_2_-)), 2.28–2.37 (m, 8H, 4(-CH_2_-)), 4.90 (s, 2H, -CH_2_-), 5.15 (s, 2H, -CH_2_-), 7.25 (d, *J* = 7.91 Hz, 1H, Ar-H), 7.32 (d, *J* = 8.12 Hz, 1H, Ar-H), 7.75 (d, *J* = 8.24 Hz, 1H, Ar-H), 8.06 (t, *J* = 7.05 Hz, 1H, Ar-H), 10.37 (s, 1H, -NH-), 10.49 (s, 1H, -NH-) ppm. M.S (EI, *m*/*z*, 70 eV): Calcd. = 438.49, Found = 438.10 [M^+^], 439.10 [M^+^ + 1], 440.10 [M^+^ + 2].

#### 3.9.5. 2,2′-(2,4-Dioxoquinazoline-1,3(2*H*,4*H*)-diyl)bis(*N*′-cyclohexylideneacetohydrazide) (**11b**)

Yield (0.25 g, 83%) as a white solid; m.p. 278–280 °C; ^1^H-NMR (500 MHz, DMSO-d_6_) δ 1.51–1.69 (m, 12H, 6(-CH_2_-)), 2.21–2.41 (m, 8H, 4(-CH_2_-)), 4.93 (d, *J* = 8.66 Hz, 2H, -CH_2_-), 5.17 (s, 2H, -CH_2_-), 7.27 (t, *J* = 7.05 Hz, 1H, -Ar-H), 7.32 (d, *J* = 8.72 Hz, 1H, Ar-H), 7.75 (t, *J* = 6.95 Hz, 1H, Ar-H), 8.06 (d, *J* = 8.59 Hz, 1H, Ar-H), 10.51 (s, 1H, -NH-), 10.75 (s, 1H, -NH-) ppm. M.S (EI, *m*/*z*, 70 eV): Calcd. = 466.54, Found = 466.15 [M^+^], 467.15 [M^+^ + 1], 468.20 [M^+^ + 2].

#### 3.9.6. 2,2′-(2,4-Dioxoquinazoline-1,3(2*H*,4*H*)-diyl)bis(*N*′-cycloheptylideneacetohydrazide) (**11c**)

Yield (0.29 g, 91%) as a white solid; m.p. 290–292 °C; ^1^H-NMR (500 MHz, DMSO-d_6_) δ 1.53–1.63 (m, 16H, 8(-CH_2_-)), 2.30–2.68 (m, 8H, 4(-CH_2_-)), 4.93 (s, 2H, -CH_2_-), 5.17 (s, 2H, -CH_2_-), 7.26 (d, *J* = 8.68 Hz, 1H, Ar-H), 7.32 (t, *J* = 7.12 Hz, 1H, Ar-H), 7.69–7.78 (m, 1H, Ar-H), 8.06 (d, *J* = 8.55 Hz, 1H, Ar-H), 10.39 (s, 1H, -NH-), 10.50 (s, 1H, -NH-) ppm. M.S (EI, *m*/*z*, 70 eV): Calcd. = 494.60, Found = 494.10 [M^+^], 495.10 [M^+^ + 1], 496.10 [M^+^ + 2].

### 3.10. General Procedure for the Synthesis of Compounds ***12a*** and ***12b***

In a 25 mL one-necked flask, a mixture of **3** (1.0 g, 3.3 mmol) and phenylisothiocyanate or phenylisocyanate (6.6 mmol) was dissolved in absolute ethanol (10 mL). The reaction mixture was heated to reflux for 10 h and the formed solid was filtered off and washed with ethanol to give **12a** or **12b**.

#### 3.10.1. 2,2′-(2,2′-(2,4-Dioxoquinazoline-1,3(2*H*,4*H*)-diyl)bis(acetyl))bis(*N*-phenylhydrazine-1-carboxamide) (**12a**)

Yield (1.5 g, 85%) as a white solid; m.p. 248–250 °C; ^1^H-NMR (400 MHz, DMSO-d_6_) δ 4.52 (s, 2H, -CH_2_-), 4.74 (s, 2H, -CH_2_-), 6.95 (t, *J* = 6.96 Hz, 1H, Ar-H), 7.15–7.25 (m, 5H, Ar-H), 7.26–7.34 (m, 5H, Ar-H), 7.48 (d, *J* = 8.84 Hz, 1H, Ar-H), 7.76 (t, *J* = 7.08 Hz, 1H, Ar-H), 8.08 (d, *J* = 8.77 Hz, 1H, Ar-H), 8.32 (br.s, 1H, -NH-), 8.58 (br.s, 1H, -NH-), 8.6 (br.s, 1H, -NH-), 9.21 (s, 1H, -NH-), 9.30 (s, 1H, -NH-), 10.09 (br.s, 1H, -NH-) ppm. ^13^C-NMR (100 MHz, DMSO-d_6_) δ 42.61, 45.03, 114.49, 115.06, 118.14, 122.23, 122.98, 127.77, 128.66, 135.28, 140.06, 150.41, 161.03, 165.87, 166.07 ppm. M.S (EI, *m*/*z*, 70 eV): Calcd. = 544.18, Found = 545.04 [M^+^ + 1].

#### 3.10.2. 2,2′-(2,2′-(2,4-Dioxoquinazoline-1,3(2*H*,4*H*)-diyl)bis(acetyl))bis(*N*-phenylhydrazine-1-carbothioamide) (**12b**)

Yield (1.8 g, 96%) as a white solid; m.p. 270–272 °C; ^1^H-NMR (400 MHz, DMSO-d_6_) δ 4.77 (s, 2H, -CH_2_-), 4.98 (s, 2H, -CH_2_-), 7.19 (d, *J* = 8.59 Hz, 2H, Ar-H), 7.34–7.38 (m, 5H, Ar-H), 7.45–7.52 (m, 5H, Ar-H), 7.79 (t, *J* = 6.96 Hz, 1H, Ar-H), 8.10 (d, *J* = 8.80 Hz, 1H, Ar-H), 9.41 (br.s, 1H, -NH-), 9.70 (br.s, 1H, -NH-), 9.76 (br.s, 1H, -NH-), 9.82 (br.s, 1H, -NH-), 10.41 (s, 1H, -NH-), 11.0 (br.s, 1H, -NH-) ppm. ^13^C-NMR (100 MHz, DMSO-d_6_) δ 42.69, 44.79, 114.49, 114.56, 115.08, 123.29, 125.11, 127.81, 128.15, 135.49, 138.93, 138.97, 140.08, 150.52, 161.04, 166.60 ppm. M.S (EI, *m*/*z*, 70 eV): Calcd. = 576.14, Found = 576.99 [M^+^ + 1].

### 3.11. 1,3-bis((5-Mercapto-4-phenyl-4H-1,2,4-triazol-3-yl)methyl)quinazoline-2,4(1H,3H)-dione (***13***)

A mixture of **12b** (0.4 g, 0.7 mmol) was added gradually with stirring to a solution of sodium hydroxide (2N, 5 mL). The mixture was heated to reflux for 11 h, after that, the reaction mixture was cooled and acidified with 2 N hydrochloric acid. The resulting precipitate was filtered off, washed with water, and the obtained compound was further purified by crystallizing from ethanol and dried to give **13** (0.3 g, 81%) as a white solid; m.p. 140–142 °C; ^1^H-NMR (400 MHz, DMSO-d_6_) δ 4.23 (s, 2H, -CH_2_-), 4.29 (s, 2H, -CH_2_-), 6.51–6.60 (m, 2H, Ar-H), 7.34–7.37 (m, 1H, Ar-H), 7.45–7.46 (m, 3H, Ar-H), 7.50–7.52 (m, 7H, Ar-H), 8.58–8.62 (m, 1H, Ar-H), 13.79 (br.s, 2H, 2(-SH)) ppm. ^13^C-NMR (100 MHz, DMSO-d_6_) δ 34.83, 44.23, 111.42, 114.48, 115.31, 117.76, 127.98, 128.43, 129.40, 132.57, 133.47, 147.80, 148.31, 149.75, 167.98, 168.73, 171.78 ppm.

### 3.12. 1,3-Bis((5-(phenylamino)-1,3,4-oxadiazol-2-yl)methyl)quinazoline-2,4(1H,3H)-dione (***14a***)

To a solution of **12b** (0.4 g, 0.7 mmol) in methanol (4 mL), mercuric oxide (0.3 g, 1.4 mmol) was added. The resulting mixture was heated under reflux conditions for 6 h. The precipitated mercuric sulfide was filtered off and washed several times with hot methanol. On cooling, the filtrate gave a solid product, which was filtered, washed with methanol, and dried to give **14a** (0.2 g, 57%) as a white solid; m.p. 160–162 °C; ^1^H-NMR (500 MHz, DMSO-d_6_) δ 5.32 (s, 2H, -CH_2_-), 5.57(s, 2H, -CH_2_-), 6.95 (t, *J* = 7.11 Hz, 1H, Ar-H), 7.28–7.31 (m, 4H, Ar-H), 7.40 (t, *J* = 6.96 Hz, 2H, Ar-H), 7.49–7.51 (m, 4H, Ar-H), 7.7 (d, *J* = 8.78 Hz, 1H, Ar-H), 7.85 (t, *J* = 7.13 Hz, 1H, Ar-H), 8.1 (d, *J* = 8.63 Hz, 1H, Ar-H), 10.47 (s, 1H, -NH-), 10.53 (s, 1H, -NH-) ppm. M.S (EI, *m*/*z*, 70 eV): Calcd. = 508.50, Found = 508.25 [M^+^], 509.20 [M^+^ + 1], 510.20 [M^+^ + 2].

### 3.13. 1,3-Bis((5-(phenylamino)-1,3,4-thiadiazol-2-yl)methyl)quinazoline-2,4(1H,3H)-dione (***14b***)

The **12b** (0.4 g, 0.7 mmol) was added gradually and carefully with stirring to ice-cooled conc. sulfuric acid (10.5 mL) over 10 min. At 0 °C, the reaction mixture was stirred for 6 h and carefully poured onto ice water. The solid separated out was filtered off, washed with water, and the obtained compound was further purified by crystallizing from ethanol and dried to give **14b** (0.25 g, 68%) as off white solid; m.p. < 360 °C; ^1^H-NMR (400 MHz, DMSO-d_6_) δ 5.43 (s, 2H, -CH_2_-), 5.62 (s, 2H, -CH_2_-), 7.38 (t, *J* = 7.07 Hz, 1H, Ar-H), 7.51-7.58 (m, 10H, Ar-H), 7.70 (d, *J* = 8.66 Hz, 1H, Ar-H), 7.84 (t, *J* = 7.02 Hz, 1H, Ar-H), 8.13 (d, *J* = 8.59 Hz, 1H, Ar-H), 10.44 (br.s, 2H, 2(-NH-)) ppm. ^13^C-NMR (100 MHz, DMSO-d_6_) δ 40.12, 42.38,114.94, 116.45, 123.63, 126.59, 128.21, 135.83, 139.34, 140.66, 141.58, 150.06, 154.28, 160.67, 165.27 ppm. M.S (EI, *m*/*z*, 70 eV): Calcd. = 540.12, Found = 541.20 [M^+^ + 1].

### 3.14. General Procedure for the Synthesis of Compounds ***15*** and ***16***

A mixture of **12a** (0.4 g, 0.8 mmol) and chloroacetonitrile or ethyl bromoacetate (1.6 mmol) in (4 mL) absolute ethanol containing anhydrous sodium acetate (0.26 g, 3.2 mmol) was heated under reflux for 12 h and 16 h, respectively. The reaction mixture was cooled, diluted with water, and allowed to stand overnight. The solid precipitate was filtered off, washed with water, and the obtained compounds were further purified by crystallizing from ethanol/acetone (8:2) and dried to give **15** and **16**, respectively.

#### 3.14.1. *N*′-(-4-Amino-3-phenyloxazol-2(3*H*)-ylidene)-2-(1-(2-(2-(-4-amino-3-phenyloxazol-2(3*H*)-ylidene)hydrazinyl)-2-oxoethyl)-2,4-dioxo-1,4-dihydroquinazolin-3(2*H*)-yl)acetohydrazide (**15**)

Yield (0.35 g, 78%) as an off white solid; m.p. 232–234 °C; ^1^H-NMR (400 MHz, DMSO-d_6_) δ 4.25 (br.s, 4H, 2(-NH_2_)), 4.52 (s, 2H, -CH_2_-), 4.74 (s, 2H, -CH_2_-), 6.95 (t, *J* = 6.93 Hz, 1H, Ar-H), 7.24–7.26 (m, 5H, Ar-H), 7.28–7.31 (m, 5H, Ar-H), 7.49 (d, *J* = 8.57 Hz, 1H, Ar-H), 7.78 (t, *J* = 7.06 Hz, 1H, Ar-H), 8.06 (d, *J* = 8.77 Hz, 1H, Ar-H), 8.79 (s, 2H, 2(-CH-)), 9.21 (s, 1H, -NH-), 9.29 (s, 1H, -NH-) ppm. ^13^C-NMR (100 MHz, DMSO-d_6_) δ 42.61, 45.03, 114.45, 115.07, 118.41, 121.76, 122.98, 127.77, 128.55, 135.28, 139.68, 140.06, 150.41, 155.99, 161.03, 165.87, 166.07 ppm. M.S (EI, *m*/*z*, 70 eV): Calcd. = 622.20, Found = 623.11 [M^+^ + 1].

#### 3.14.2. 2-(2,4-Dioxo-1-(2-oxo-2-(2-(-4-oxo-3-phenyloxazolidin-2-ylidene)hydrazinyl)ethyl)-1,4-dihydroquinazolin-3(2*H*)-yl)-*N*′-(-4-oxo-3-phenyloxazolidin-2-ylidene)acetohydrazide (**16**)

Yield (0.3 g, 67%) as a white solid; m.p. 234–236 °C; ^1^H-NMR (400 MHz, DMSO-d_6_) δ 4.22 (s, 2H, -CH_2_-), 4.28 (s, 2H, -CH_2_-), 4.52 (s, 2 H, -OCH_2_-), 4.74 (s, 2H, -OCH_2_-), 6.94 (t, *J* = 7.03 Hz, 1H, Ar-H), 7.22–7.26 (m, 5H, Ar-H), 7.31–7.34 (m, 5H, Ar-H), 7.49 (d, *J* = 8.76 Hz, 1H, Ar-H), 7.78 (t, *J* = 6.78 Hz, 1H, Ar-H), 8.06 (d, *J* = 8.57 Hz, 1H, Ar-H), 9.21 (s, 1H, -NH-), 9.29 (s, 1H, -NH-) ppm. ^13^C-NMR (100 MHz, DMSO-d_6_) δ 42.60, 45.02, 114.64, 115.06, 118.40, 121.73, 122.97, 127.76, 128.58, 135.27, 139.70, 140.05, 150.44, 156, 160.96, 161.02, 165.99, 166.15, 169.84 ppm. M.S (EI, *m*/*z*, 70 eV): Calcd. = 624.17, Found = 625.06 [M^+^ + 1].

## 4. Biological Methods

### Antibacterial Assay

The stock cultures of pathogens used in this study were maintained on nutrient agar slants at 4°. The reference antibacterial drugs ampicillin and vancomycin were evaluated for their antibacterial and antifungal activities and compared with the synthesized compounds. Seventy microliters of bacterial and yeast cells (10^6^ CFU/mL) of each pathogen were spread on the nutrient agar plates. The wells (6 mm diameter) were dug in the inoculated agar plates and 100 µL of the tested compound in DMSO (100 mg/mL) was added to the wells. The reference antibiotic disks (10 µg/disk of ampicillin and 30 µg/disk of vancomycin) were potted onto the surface of agar inoculated plates. Before incubation, the plates were allowed to stand for 2 h at 4 °C to allow for diffusion. Subsequently, the plates were incubated for 24 h at 37 °C, except for the yeast strain, which was incubated for 24 h at 28 °C. Next, we applied the measurement of the diameter of the inhibition zone (mm) and three replicates were averaged [58].

MIC calculation: The MIC was defined as the concentration at which the bacteria and yeast do not show visible growth with respect to the positive control. Serial dilutions were prepared of the tested compounds dissolved in DMSO. In each well of the 96 well microtiter plate, a 150 μL of double strength Mueller Hinton broth medium was loaded followed by 150 μL of the 2-fold appropriate concentration, and mixed well to obtain the final concentration. Overnight broth cultures of the tested bacterial and yeast strains prepared as an inoculum of 5% (*V*/*V*) (OD = 0.5 McFarland standard) was inoculated into the respective wells. For the positive-growth control, the same inoculum size of each test strain was inoculated in wells that did not contain any of the tested compounds. The DMSO solution was tested as the negative control. The plates were statically incubated at 37 °C for 24 h. A total of 30 μL of resazurin solution (0.18%) was added to each well to act as an electron acceptor and was reduced to a pink, red, or purple resorufin colored product by active microorganisms (i.e., the inhibition of bacterial growth was appearing as a dark blue well and the presence of growth was detected by the presence of a pink, red, or purple color).

## 5. Conclusions

In conclusion, a series of novel quinazoline-2,4(1*H*,3*H*)-dione derivatives were designed and synthesized by installing different moieties into in the 1- and 3-positions of the quinazoline nucleus and their structures were determined based on the spectral data. The synthesized compounds generally showed moderate or no activity against the tested strains *Staphylococcus aurous*, *Listeria monocytogenes*, *Escherichia coli*, *Salmonella typhi*, and *Candida albicans* in comparison with the reference drugs ampicillin and vancomycin. Among the tested compounds, 1,3-bis((5-mercapto-4-phenyl-4*H*-1,2,4-triazol-3-yl)methyl)quinazoline-2,4(1*H*,3*H*)-dione (**13**) and *N*′-(-4-amino-3-phenyloxazol-2(3*H*)-ylidene)-2-(1-(2-(2-((*E*)-4-amino-3-phenyloxazol-2(3*H*) ylidene)hydrazinyl)-2-oxoethyl)-2,4-dioxo-1,4-dihydroquinazolin-3(2*H*)-yl)acetohydrazide (**15**) generally exhibited better and broad bioactive spectrum against Gram-positive and Gram-negative strains. For our further studies, derivatives **13** and **15** were selected as promising leads to synthesize more selective derivatives to generate more effective antibacterial agents.

## Figures and Tables

**Table 1:** The antimicrobial activity as the inhibition zone value (IZ mm) and minimum inhibitory concentration (MICs mg/mL) for the synthesized compounds against *Staphylococcus aurous*, *Listeria monocytogenes*, *Escherichia coli*, *Salmonella typhi*, and *Candida albicans* strains.

Sample	*S. aureus*	*L. monocytogenes*	*E. coli*	*S. typhi*	*C. albicans*
	MICs mg/mL, (IZ mm)	MICs mg/mL, (IZ mm)	MICs mg/mL, (IZ mm)	MICs mg/mL, (IZ mm)	MICs mg/mL, (IZ mm)
Control: DMSO	0.0	0.0	0.0	0.0	0.0
**3**	>100	>100	>100	>100	80 (11)
**7**	>100	>100	>100	>100	>100
**8a**	>100	>100	>100	>100	>100
**8b**	>100	>100	>100	>100	>100
**9a**	>100	>100	>100	>100	>100
**9b**	>100	>100	>100	>100	80 (11)
**10a**	>100	>100	>100	>100	>100
**10b**	>100	>100	>100	>100	>100
**10c**	>100	>100	>100	>100	>100
**11a**	>100	>100	>100	>100	>100
**11b**	>100	>100	>100	>100	>100
**11c**	>100	>100	>100	>100	>100
**12b**	>100	>100	>100	>100	>100
**13**	90 (9)	>100	65 (15)	>100	80 (11)
**14a**	75 (12)	>100	>100	>100	75 (12)
**14b**	70 (13)	>100	>100	>100	90 (9)
**15**	80 (11)	>100	75 (12)	>100	77 (10)
**16**	>100	>100	>100	>100	>100
Ampicillin	(25)	(20)	(16)	(19)	(9)
Vancomycin	(14)	(15)	(15)	(17)	(15)

## Data Availability

Not applicable.

## Supplementary Materials

The following supporting information can be downloaded at: https://www.mdpi.com/article/10.3390/molecules27123853/s1; ^1^H- and ^13^C-NMR spectral data of compounds **3**–**16**.

## Sample Availability

Samples of the compounds are available from the authors.
