# Supplementary material for: Discovery of Quinazoline-2,4(1H,3H)-Dione Derivatives as Potential Antibacterial Agent: Design, Synthesis, and Their Antibacterial Activity
Source: Molecules. 2022 Jun 15;27(12):3853. doi: 10.3390/molecules27123853 (PMC9228007; doi:10.3390/molecules27123853)
Supplement: Supplementary file 1 [file molecules-27-03853-s001.zip › molecules-1738610-supplementary.pdf]

molecules

Supplementary Materials

Discovery of Quinazoline-2,4(1*H*,3*H*)-Dione Derivatives as Potential Antibacterial Agent: Design, Synthesis, and Their Antibacterial Activity

Nader M. Boshta [1,*,†], Farag A. El-Essawy [2], Mohammed B. Alshammari [3], Safaa G. Noreldein [1] and Osama M. Darwesh [4]

[1] Chemistry Department, Faculty of Science, Menoufia University, Shebin El-Koam 32511, Egypt; chemsafaagoda@gmail.com
[2] Preparatory Year Deanship, Basic Science Department, Prince Sattam Bin Abdulaziz University, 151 Alkharj 11942, Saudi Arabia; f.elessawy@psau.edu.sa
[3] College of Sciences and Humanities, Prince Sattam Bin Abdulaziz University, 151 Alkharj 11942, Saudi Arabia; m.alshammari@psau.edu.sa
[4] Environmental Microbiology and Nanotechnology group, Agricultural Microbiology Department, National Research Centre, Cairo 12622, Egypt; darweshosama@yahoo.com
* Correspondence: nboshta@uni-bonn.de; Tel.: +20-10-6604-7824; Fax: +20-48-223-5689
† Current address: Pharmaceutical Institute, Pharmaceutical Chemistry I, University of Bonn, An der Immenburg 4, D-53121 Bonn, Germany.

Comp.3

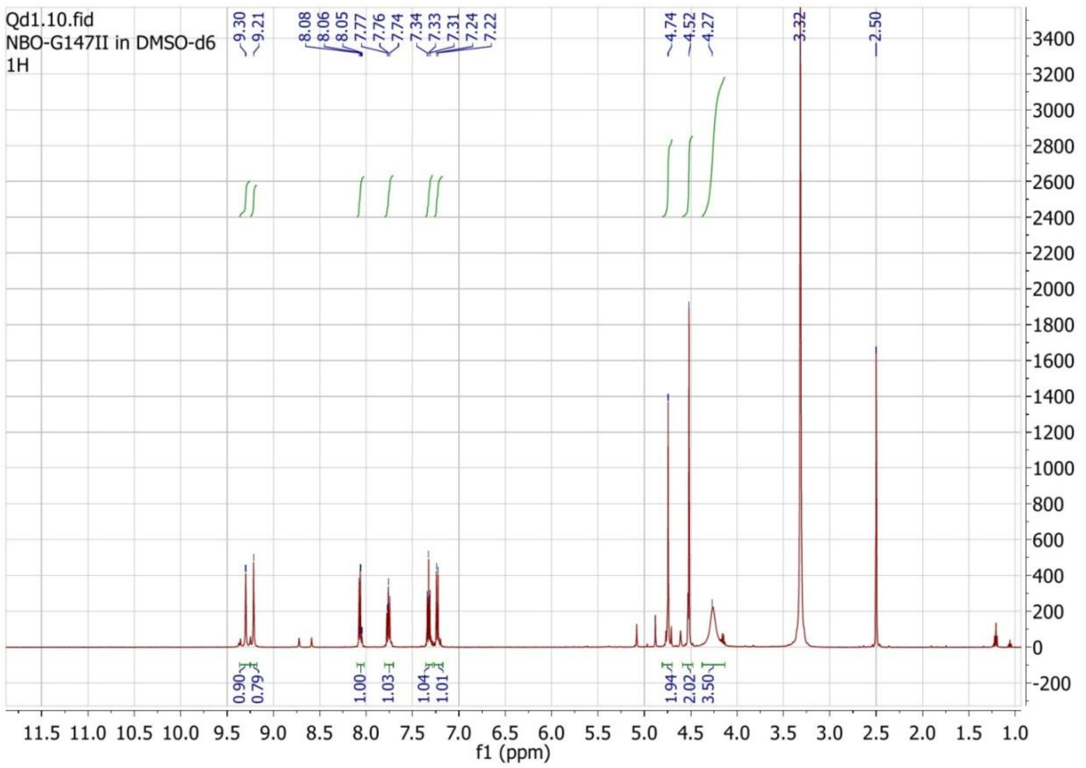
Qd1.10.fid
NBO-G147II in DMSO-d6
1H
9.30
9.21
8.08
8.06
8.05
7.77
7.76
7.74
7.34
7.33
7.31
7.24
7.22
4.74
4.52
4.27
3.32
2.50
0.90
0.79
1.00
1.03
1.04
1.01
1.94
2.02
3.50
f1 (ppm)

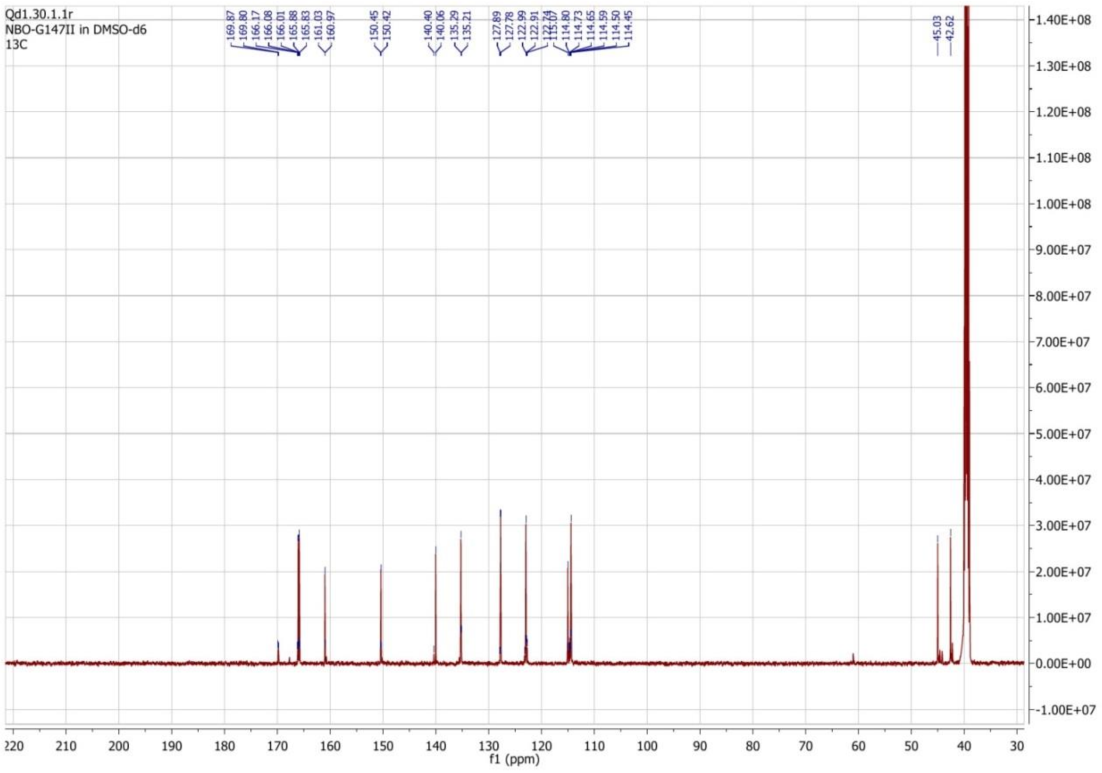
Qd1.30.1.1r
NBO-G147II in DMSO-d6
13C
169.87
169.80
166.17
166.08
166.01
165.88
165.83
161.03
160.97
150.45
150.42
140.40
140.06
135.29
135.21
127.89
127.78
122.99
122.91
122.74
115.07
114.80
114.73
114.65
114.59
114.50
114.45
45.03
42.62
f1 (ppm)

Comp.4

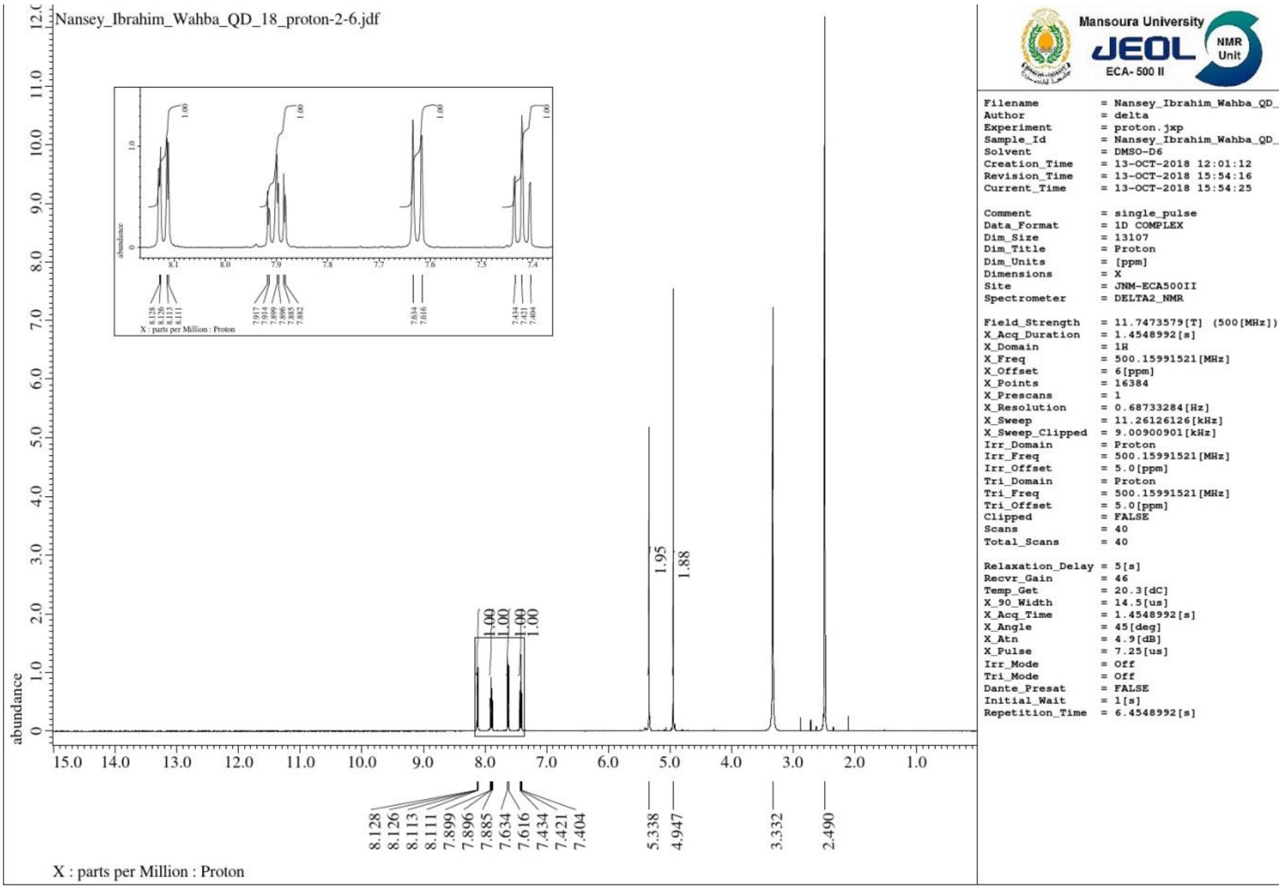

Nansey_Ibrahim_Wahba_QD_18_proton-2-6.jdf
Mansoura University
JEOL
NMR Unit
ECA- 500 II
Filename = Nansey_Ibrahim_Wahba_QD_
Author = delta
Experiment = proton.jxp
Sample_Id = Nansey_Ibrahim_Wahba_QD_
Solvent = DMSO-D6
Creation_Time = 13-OCT-2018 12:01:12
Revision_Time = 13-OCT-2018 15:54:16
Current_Time = 13-OCT-2018 15:54:25
Comment = single_pulse
Data_Format = 1D COMPLEX
Dim_Size = 13107
Dim_Title = Proton
Dim_Units = [ppm]
Dimensions = X
Site = JNM-ECA500II
Spectrometer = DELTA2_NMR
Field_Strength = 11.7473579[T] (500[MHz])
X_Acq_Duration = 1.4548992[s]
X_Domain = 1H
X_Freq = 500.15991521[MHz]
X_Offset = 6[ppm]
X_Points = 16384
X_Prescans = 1
X_Resolution = 0.68733284[Hz]
X_Sweep = 11.26126126[kHz]
X_Sweep_Clipped = 9.00900901[kHz]
Irr_Domain = Proton
Irr_Freq = 500.15991521[MHz]
Irr_Offset = 5.0[ppm]
Tri_Domain = Proton
Tri_Freq = 500.15991521[MHz]
Tri_Offset = 5.0[ppm]
Clipped = FALSE
Scans = 40
Total_Scans = 40
Relaxation_Delay = 5[s]
Recvr_Gain = 46
Temp_Get = 20.3[dC]
X_90_Width = 14.5[us]
X_Acq_Time = 1.4548992[s]
X_Angle = 45[deg]
X_Atn = 4.9[dB]
X_Pulse = 7.25[us]
Irr_Mode = Off
Tri_Mode = Off
Dante_Presat = FALSE
Initial_Wait = 1[s]
Repetition_Time = 6.4548992[s]
abundance
X : parts per Million : Proton
8.128
8.126
8.113
8.111
7.917
7.914
7.899
7.896
7.885
7.882
7.634
7.616
7.434
7.421
7.404
5.338
4.947
3.332
2.490
1.00
1.00
1.00
1.00
1.95
1.88

Comp.5

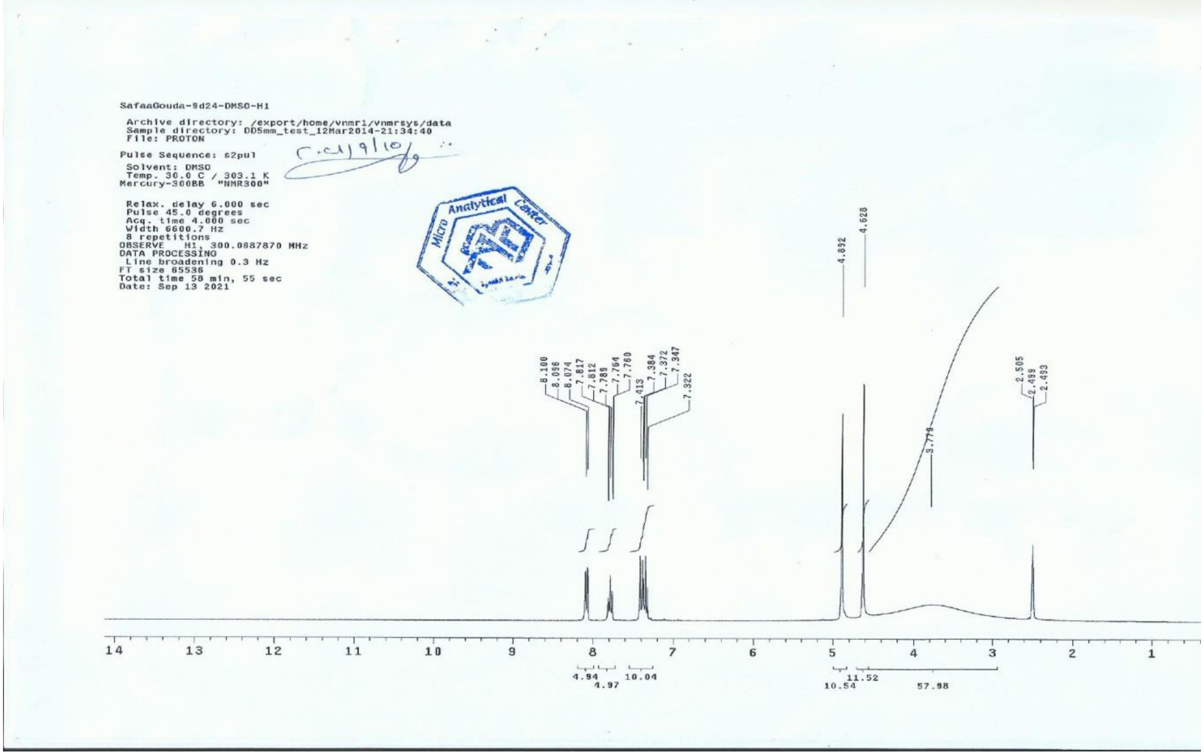
SafaaGouda-Sd24-DMSO-H1
Archive directory: /export/home/vnmr1/vnmrsys/data
Sample directory: DD5mm_test_12Mar2014-21:34:40
File: PROTON
Pulse Sequence: s2pul
Solvent: DMSO
Temp. 30.0 C / 303.1 K
Mercury-300BB "NMR300"
Relax. delay 6.000 sec
Pulse 45.0 degrees
Acq. time 4.000 sec
Width 6600.7 Hz
8 repetitions
OBSERVE H1, 300.0887870 MHz
DATA PROCESSING
Line broadening 0.3 Hz
FT size 65536
Total time 58 min, 55 sec
Date: Sep 13 2021
Micro Analytical Center
8.100
8.096
8.074
7.817
7.812
7.789
7.764
7.760
7.413
7.384
7.372
7.347
7.322
4.892
4.628
3.778
2.505
2.499
2.493
14
13
12
11
10
9
8
7
6
5
4
3
2
1
4.94
4.97
10.04
10.54
11.52
57.98

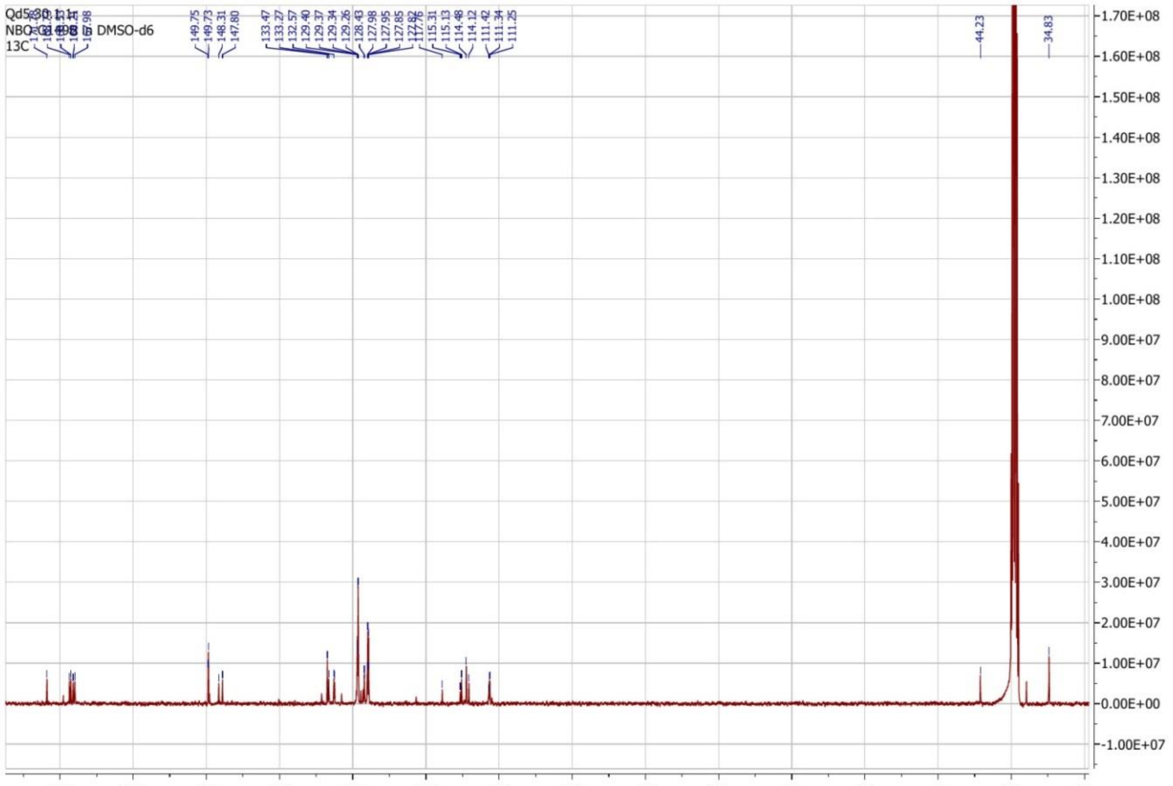
Qd5-30.1.1.fid
NBO-C14DB in DMSO-d6
13C
149.75
149.73
148.31
147.80
133.47
133.27
132.57
129.40
129.37
129.34
129.26
128.43
127.98
127.95
127.85
115.31
115.13
114.48
114.12
111.42
111.34
111.25
44.23
34.83
1.70E+08
1.60E+08
1.50E+08
1.40E+08
1.30E+08
1.20E+08
1.10E+08
1.00E+08
9.00E+07
8.00E+07
7.00E+07
6.00E+07
5.00E+07
4.00E+07
3.00E+07
2.00E+07
1.00E+07
0.00E+00
-1.00E+07
170
160
150
140
130
120
110
100
90
80
70
60
50
40
30
f1 (ppm)

Comp.6

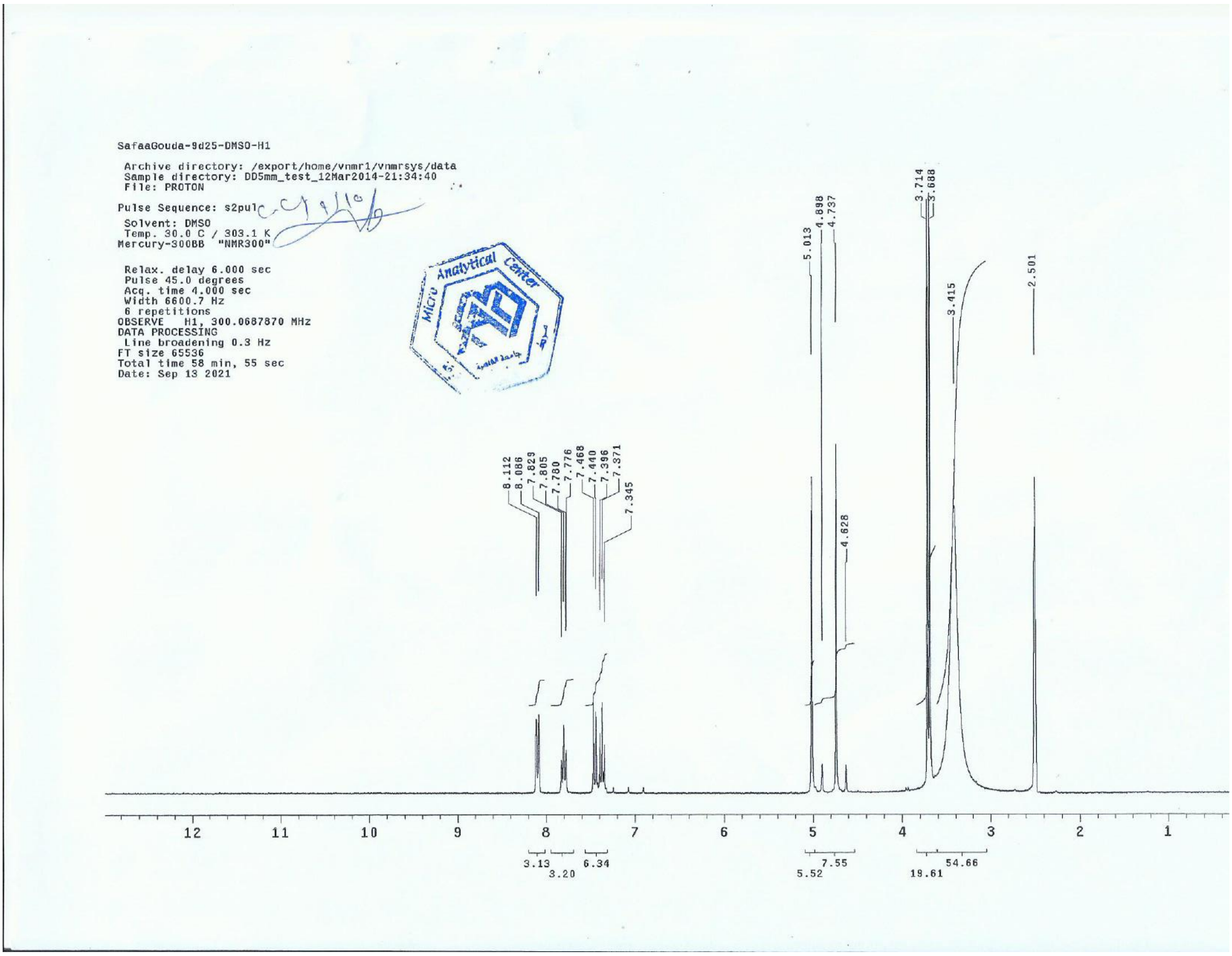

SafaaGouda-9d25-DMSO-H1
Archive directory: /export/home/vnmr1/vnmrsys/data
Sample directory: DD5mm_test_12Mar2014-21:34:40
File: PROTON
Pulse Sequence: s2pul
Solvent: DMSO
Temp. 30.0 C / 303.1 K
Mercury-300BB "NMR300"
Relax. delay 6.000 sec
Pulse 45.0 degrees
Acq. time 4.000 sec
Width 6600.7 Hz
6 repetitions
OBSERVE H1, 300.0687870 MHz
DATA PROCESSING
Line broadening 0.3 Hz
FT size 65536
Total time 58 min, 55 sec
Date: Sep 13 2021
Micro Analytical Center
8.112
8.086
7.829
7.805
7.780
7.776
7.468
7.440
7.396
7.371
7.345
5.013
4.898
4.737
4.628
3.714
3.688
3.415
2.501
12
11
10
9
8
7
6
5
4
3
2
1
3.13
3.20
6.34
5.52
7.55
19.61
54.66

Comp.7

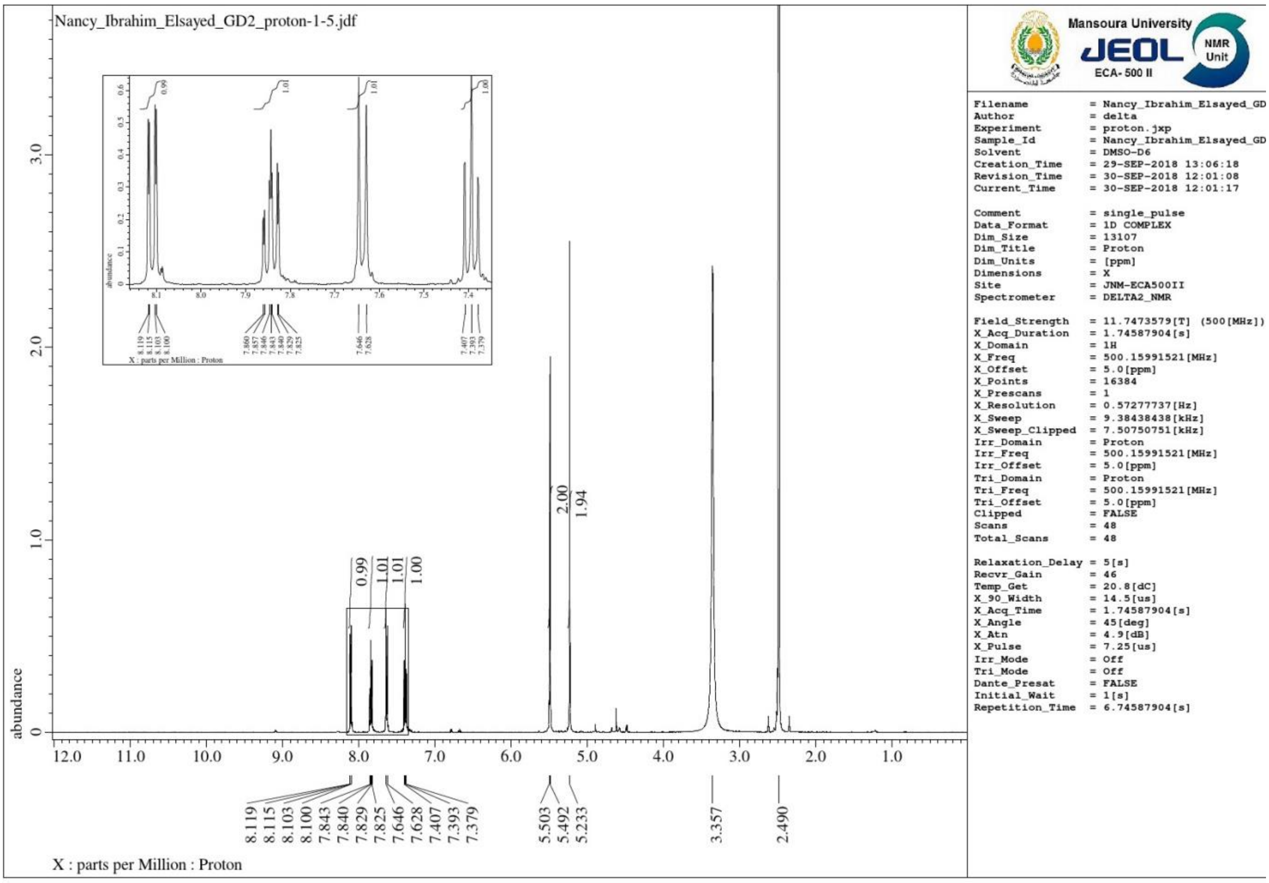

Nancy_Ibrahim_Elsayed_GD2_proton-1-5.jdf
abundance
X : parts per Million : Proton
8.119
8.115
8.103
8.100
7.843
7.840
7.829
7.825
7.646
7.628
7.407
7.393
7.379
5.503
5.492
5.233
3.357
2.490
0.99
1.01
1.01
1.00
2.00
1.94
Mansoura University
JEOL
NMR Unit
ECA- 500 II
Filename = Nancy_Ibrahim_Elsayed_GD
Author = delta
Experiment = proton.jxp
Sample_Id = Nancy_Ibrahim_Elsayed_GD
Solvent = DMSO-D6
Creation_Time = 29-SEP-2018 13:06:18
Revision_Time = 30-SEP-2018 12:01:08
Current_Time = 30-SEP-2018 12:01:17
Comment = single_pulse
Data_Format = 1D COMPLEX
Dim_Size = 13107
Dim_Title = Proton
Dim_Units = [ppm]
Dimensions = X
Site = JNM-ECA500II
Spectrometer = DELTA2_NMR
Field_Strength = 11.7473579[T] (500[MHz])
X_Acq_Duration = 1.74587904[s]
X_Domain = 1H
X_Freq = 500.15991521[MHz]
X_Offset = 5.0[ppm]
X_Points = 16384
X_Prescans = 1
X_Resolution = 0.57277737[Hz]
X_Sweep = 9.38438438[kHz]
X_Sweep_Clipped = 7.50750751[kHz]
Irr_Domain = Proton
Irr_Freq = 500.15991521[MHz]
Irr_Offset = 5.0[ppm]
Tri_Domain = Proton
Tri_Freq = 500.15991521[MHz]
Tri_Offset = 5.0[ppm]
Clipped = FALSE
Scans = 48
Total_Scans = 48
Relaxation_Delay = 5[s]
Recvr_Gain = 46
Temp_Get = 20.8[dC]
X_90_Width = 14.5[us]
X_Acq_Time = 1.74587904[s]
X_Angle = 45[deg]
X_Atn = 4.9[dB]
X_Pulse = 7.25[us]
Irr_Mode = Off
Tri_Mode = Off
Dante_Presat = FALSE
Initial_Wait = 1[s]
Repetition_Time = 6.74587904[s]

Comp.8a

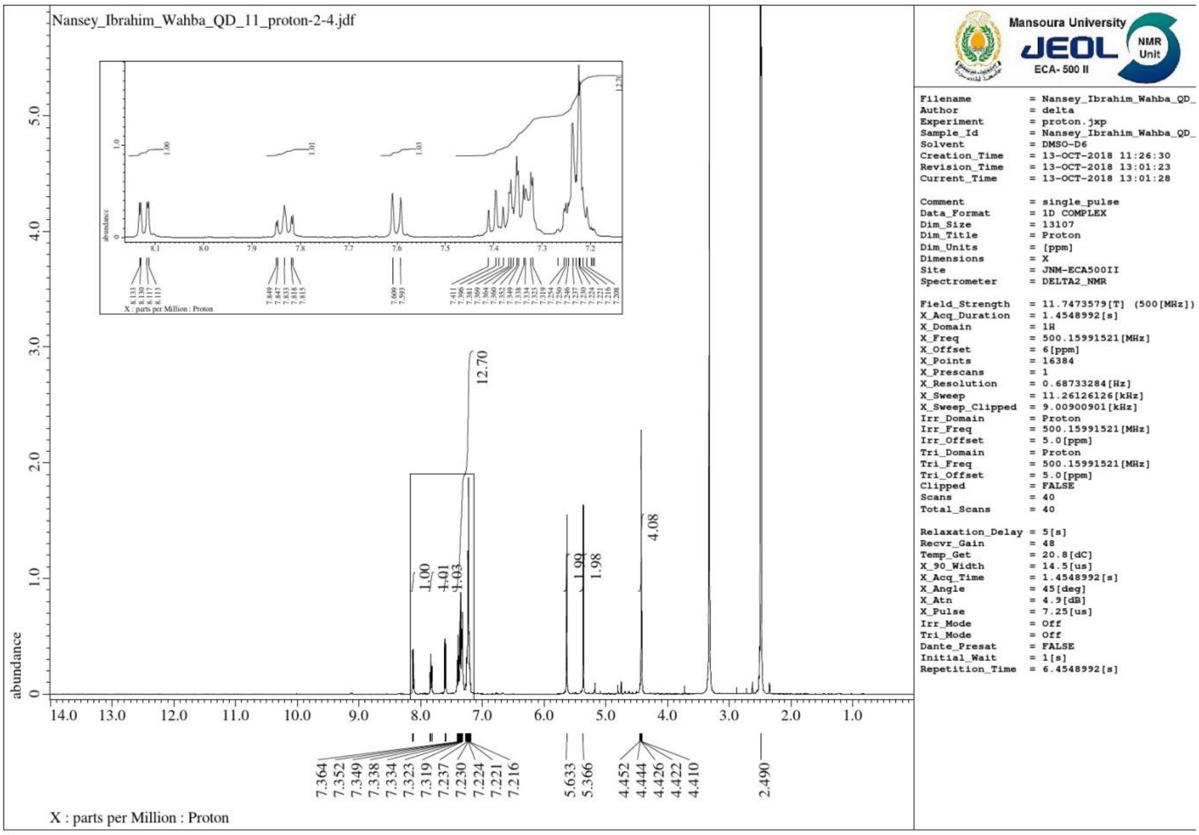
Nansey_Ibrahim_Wahba_QD_11_proton-2-4.jdf
Mansoura University
JEOL
NMR Unit
ECA-500 II
Filename = Nansey_Ibrahim_Wahba_QD_
Author = delta
Experiment = proton.jxp
Sample_Id = Nansey_Ibrahim_Wahba_QD_
Solvent = DMSO-D6
Creation_Time = 13-OCT-2018 11:26:30
Revision_Time = 13-OCT-2018 13:01:23
Current_Time = 13-OCT-2018 13:01:28
Comment = single_pulse
Data_Format = 1D COMPLEX
Dim_Size = 13107
Dim_Title = Proton
Dim_Units = [ppm]
Dimensions = X
Site = JNM-ECA500II
Spectrometer = DELTA2_NMR
Field_Strength = 11.7473579[T] (500[MHz])
X_Acq_Duration = 1.4548992[s]
X_Domain = 1H
X_Freq = 500.15991521[MHz]
X_Offset = 6[ppm]
X_Points = 16384
X_Prescans = 1
X_Resolution = 0.68733284[Hz]
X_Sweep = 11.26126126[kHz]
X_Sweep_Clipped = 9.00900901[kHz]
Irr_Domain = Proton
Irr_Freq = 500.15991521[MHz]
Irr_Offset = 5.0[ppm]
Tri_Domain = Proton
Tri_Freq = 500.15991521[MHz]
Tri_Offset = 5.0[ppm]
Clipped = FALSE
Scans = 40
Total_Scans = 40
Relaxation_Delay = 5[s]
Recvr_Gain = 48
Temp_Get = 20.8[dC]
X_90_Width = 14.5[us]
X_Acq_Time = 1.4548992[s]
X_Angle = 45[deg]
X_Atn = 4.9[dB]
X_Pulse = 7.25[us]
Irr_Mode = Off
Tri_Mode = Off
Dante_Presat = FALSE
Initial_Wait = 1[s]
Repetition_Time = 6.4548992[s]
abundance
12.70
1.00
1.01
1.03
1.99
1.98
4.08
7.364
7.352
7.349
7.338
7.334
7.323
7.319
7.237
7.230
7.224
7.221
7.216
5.633
5.366
4.452
4.444
4.426
4.422
4.410
2.490
X : parts per Million : Proton

Comp.8b

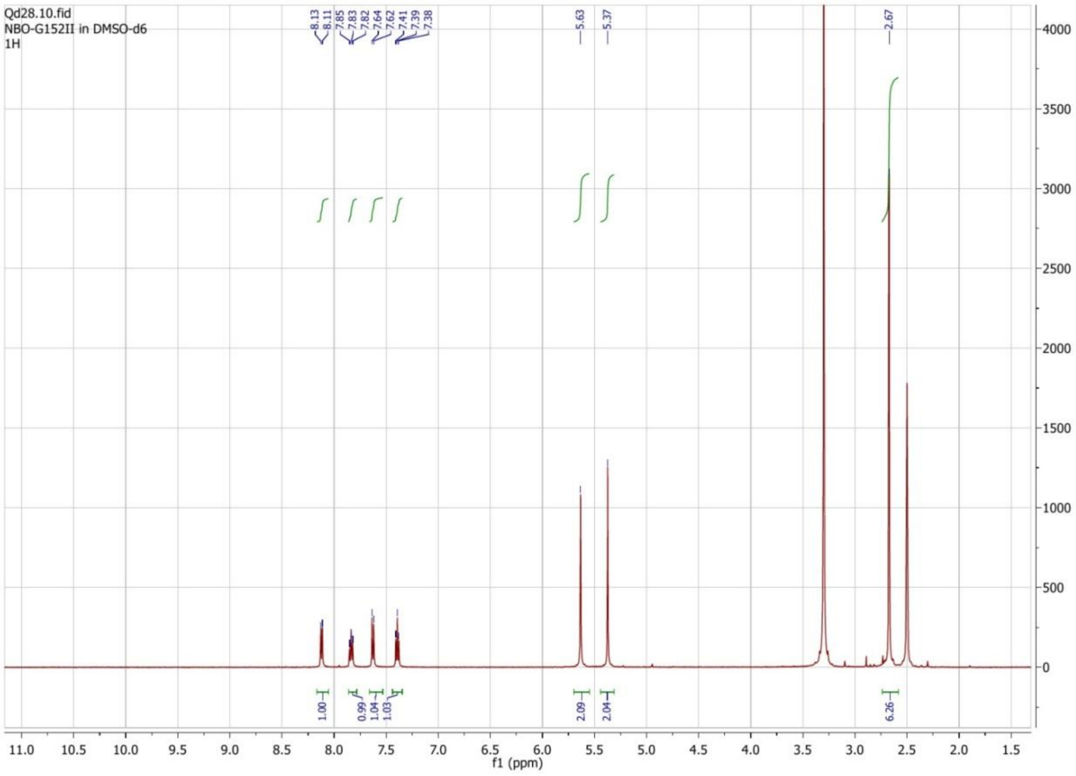
Qd28.10.fid
NBO-G152II in DMSO-d6
1H
8.13
8.11
7.85
7.83
7.82
7.64
7.62
7.41
7.39
7.38
5.63
5.37
2.67
1.00
0.99
1.04
1.03
2.09
2.04
6.26
f1 (ppm)

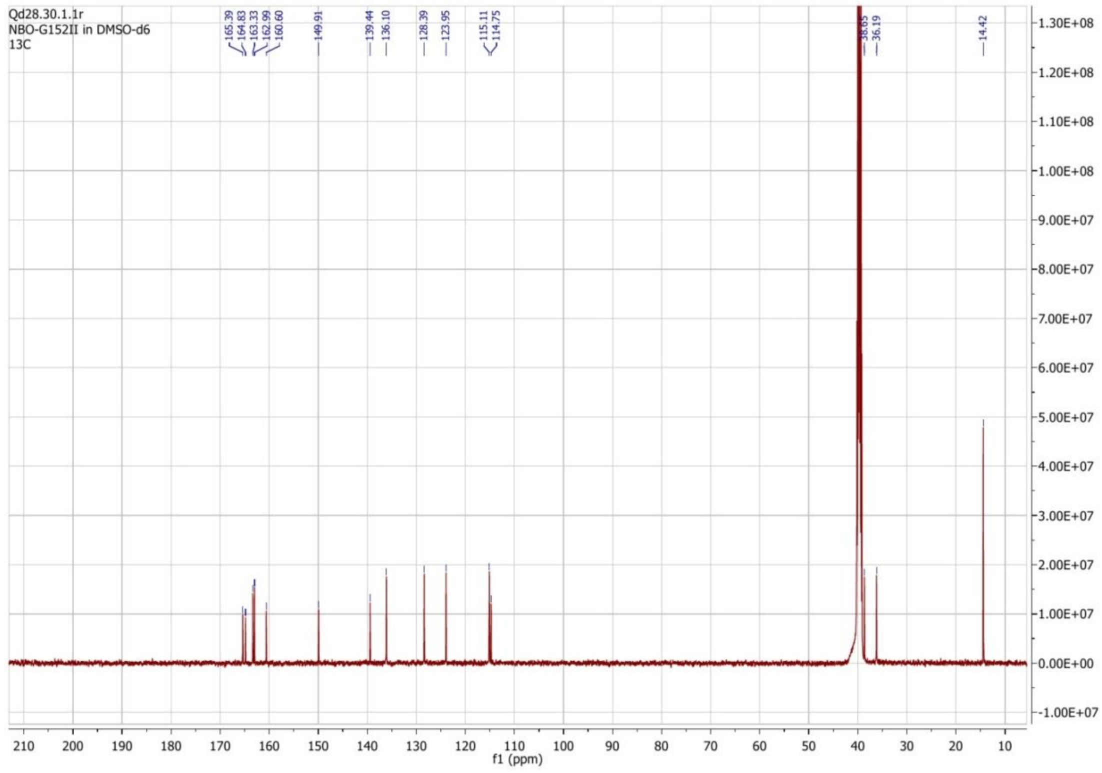
Qd28.30.1.1r
NBO-G152II in DMSO-d6
13C
165.39
164.83
163.33
162.99
160.60
149.91
139.44
136.10
128.39
123.95
115.11
114.75
38.65
36.19
14.42
f1 (ppm)

Comp.9b

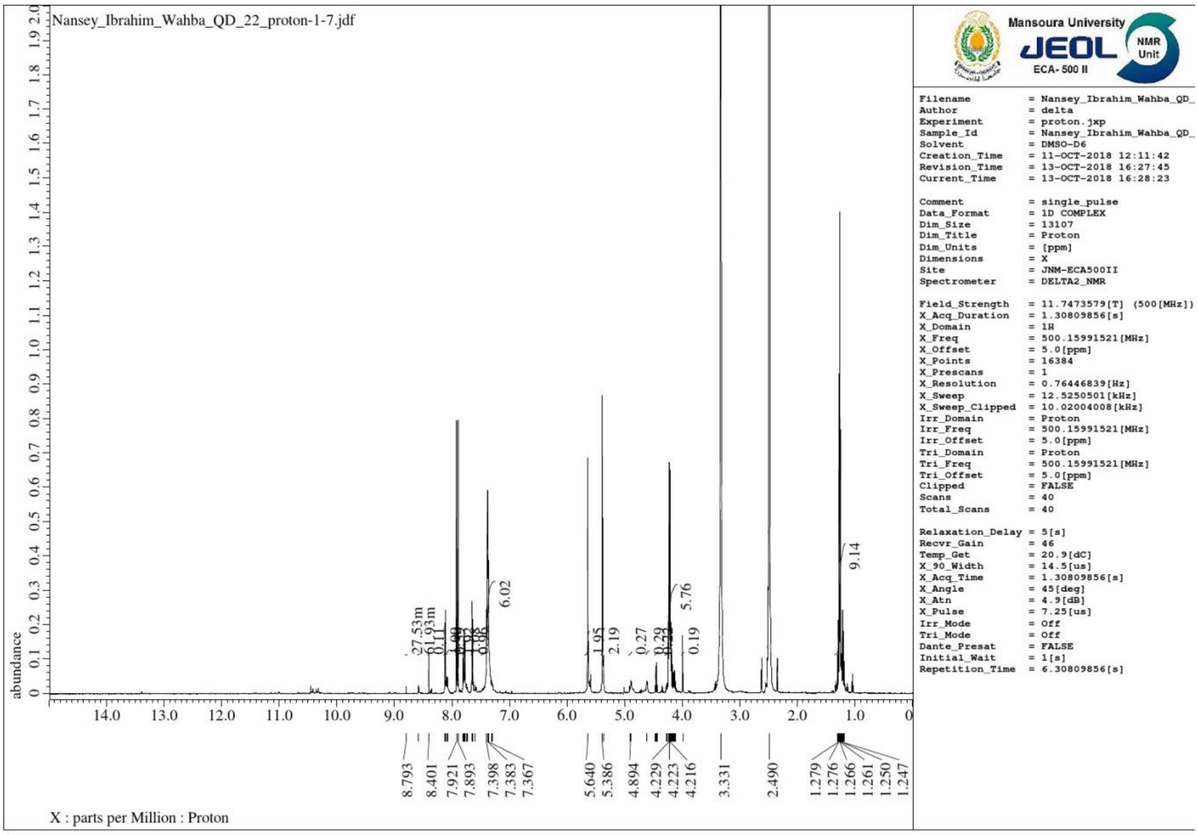

Nansey_Ibrahim_Wahba_QD_22_proton-1-7.jdf
Mansoura University
JEOL
ECA- 500 II
NMR Unit
Filename = Nansey_Ibrahim_Wahba_QD_
Author = delta
Experiment = proton.jxp
Sample_Id = Nansey_Ibrahim_Wahba_QD_
Solvent = DMSO-D6
Creation_Time = 11-OCT-2018 12:11:42
Revision_Time = 13-OCT-2018 16:27:45
Current_Time = 13-OCT-2018 16:28:23
Comment = single_pulse
Data_Format = 1D COMPLEX
Dim_Size = 13107
Dim_Title = Proton
Dim_Units = [ppm]
Dimensions = X
Site = JNM-ECA500II
Spectrometer = DELTA2_NMR
Field_Strength = 11.7473579[T] (500[MHz])
X_Acq_Duration = 1.30809856[s]
X_Domain = 1H
X_Freq = 500.15991521[MHz]
X_Offset = 5.0[ppm]
X_Points = 16384
X_Prescans = 1
X_Resolution = 0.76446839[Hz]
X_Sweep = 12.5250501[kHz]
X_Sweep_Clipped = 10.02004008[kHz]
Irr_Domain = Proton
Irr_Freq = 500.15991521[MHz]
Irr_Offset = 5.0[ppm]
Tri_Domain = Proton
Tri_Freq = 500.15991521[MHz]
Tri_Offset = 5.0[ppm]
Clipped = FALSE
Scans = 40
Total_Scans = 40
Relaxation_Delay = 5[s]
Recvr_Gain = 46
Temp_Get = 20.9[dC]
X_90_Width = 14.5[us]
X_Acq_Time = 1.30809856[s]
X_Angle = 45[deg]
X_Atn = 4.9[dB]
X_Pulse = 7.25[us]
Irr_Mode = Off
Tri_Mode = Off
Dante_Presat = FALSE
Initial_Wait = 1[s]
Repetition_Time = 6.30809856[s]
abundance
X : parts per Million : Proton
8.793 8.401 7.921 7.893 7.398 7.383 7.367 5.640 5.386 4.894 4.229 4.223 4.216 3.331 2.490 1.279 1.276 1.266 1.261 1.250 1.247
27.53m 61.93m 0.11 6.02 1.95 2.19 0.27 0.29 5.76 0.19 9.14

Comp.10a

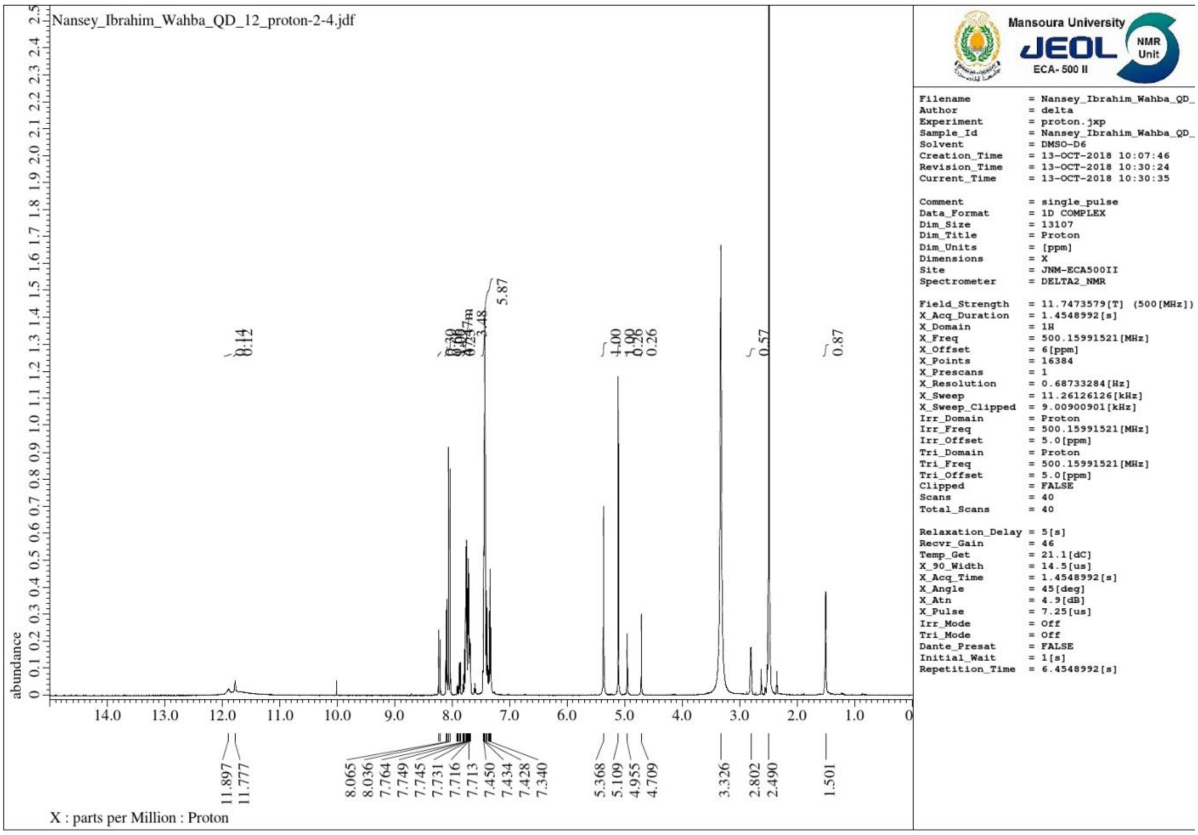

Comp.10b

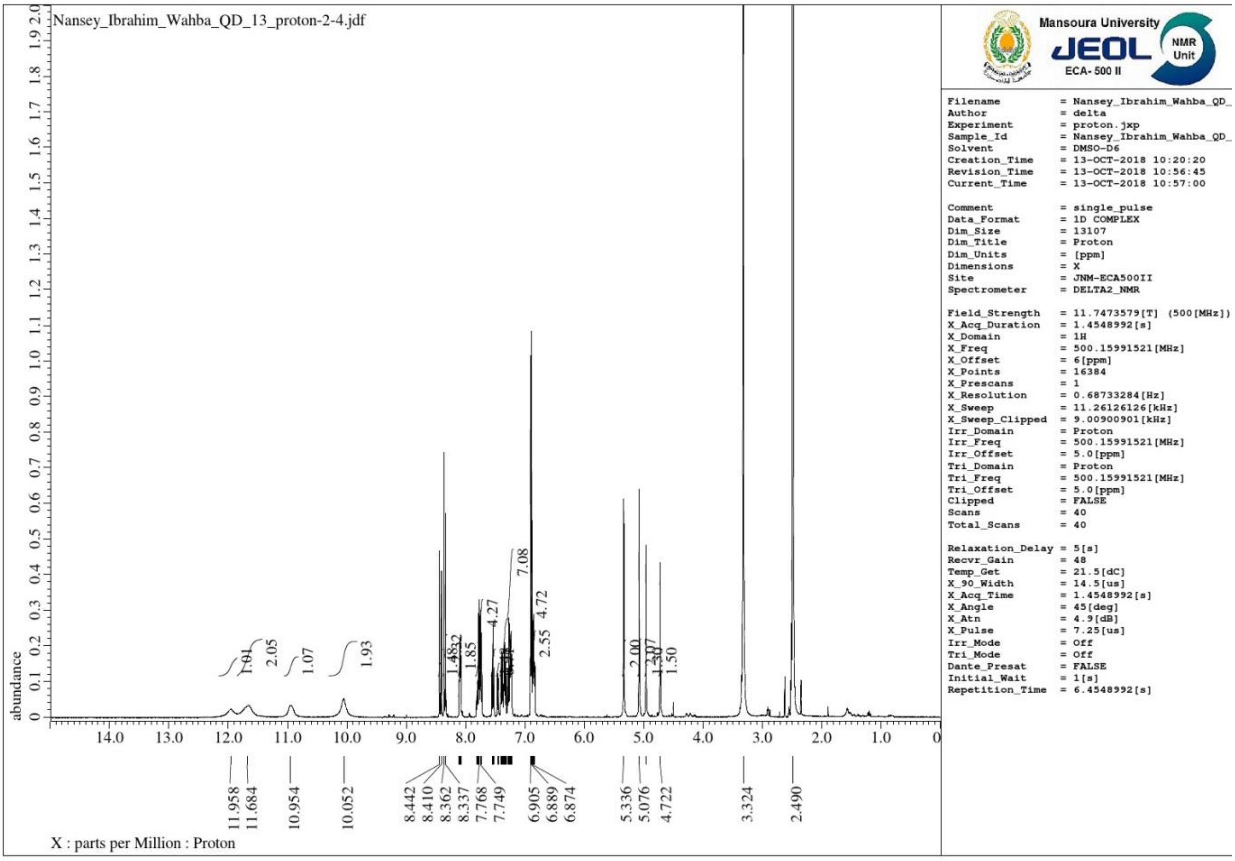

Nansey_Ibrahim_Wahba_QD_13_proton-2-4.jdf
Mansoura University
JEOL
NMR Unit
ECA- 500 II
Filename = Nansey_Ibrahim_Wahba_QD_
Author = delta
Experiment = proton.jxp
Sample_Id = Nansey_Ibrahim_Wahba_QD_
Solvent = DMSO-D6
Creation_Time = 13-OCT-2018 10:20:20
Revision_Time = 13-OCT-2018 10:56:45
Current_Time = 13-OCT-2018 10:57:00
Comment = single_pulse
Data_Format = 1D COMPLEX
Dim_Size = 13107
Dim_Title = Proton
Dim_Units = [ppm]
Dimensions = X
Site = JNM-ECA500II
Spectrometer = DELTA2_NMR
Field_Strength = 11.7473579[T] (500[MHz])
X_Acq_Duration = 1.4548992[s]
X_Domain = 1H
X_Freq = 500.15991521[MHz]
X_Offset = 6[ppm]
X_Points = 16384
X_Prescans = 1
X_Resolution = 0.68733284[Hz]
X_Sweep = 11.26126126[kHz]
X_Sweep_Clipped = 9.00900901[kHz]
Irr_Domain = Proton
Irr_Freq = 500.15991521[MHz]
Irr_Offset = 5.0[ppm]
Tri_Domain = Proton
Tri_Freq = 500.15991521[MHz]
Tri_Offset = 5.0[ppm]
Clipped = FALSE
Scans = 40
Total_Scans = 40
Relaxation_Delay = 5[s]
Recvr_Gain = 48
Temp_Get = 21.5[dC]
X_90_Width = 14.5[us]
X_Acq_Time = 1.4548992[s]
X_Angle = 45[deg]
X_Atn = 4.9[dB]
X_Pulse = 7.25[us]
Irr_Mode = Off
Tri_Mode = Off
Dante_Presat = FALSE
Initial_Wait = 1[s]
Repetition_Time = 6.4548992[s]
abundance
0 0.1 0.2 0.3 0.4 0.5 0.6 0.7 0.8 0.9 1.0 1.1 1.2 1.3 1.4 1.5 1.6 1.7 1.8 1.9 2.0
14.0 13.0 12.0 11.0 10.0 9.0 8.0 7.0 6.0 5.0 4.0 3.0 2.0 1.0 0
1.01 2.05 1.07 1.93 1.48 1.85 4.27 7.08 4.72 2.55 2.00 2.07 1.50
11.958 11.684 10.954 10.052 8.442 8.410 8.362 8.337 7.768 7.749 6.905 6.889 6.874 5.336 5.076 4.722 3.324 2.490
X : parts per Million : Proton

Comp.10c

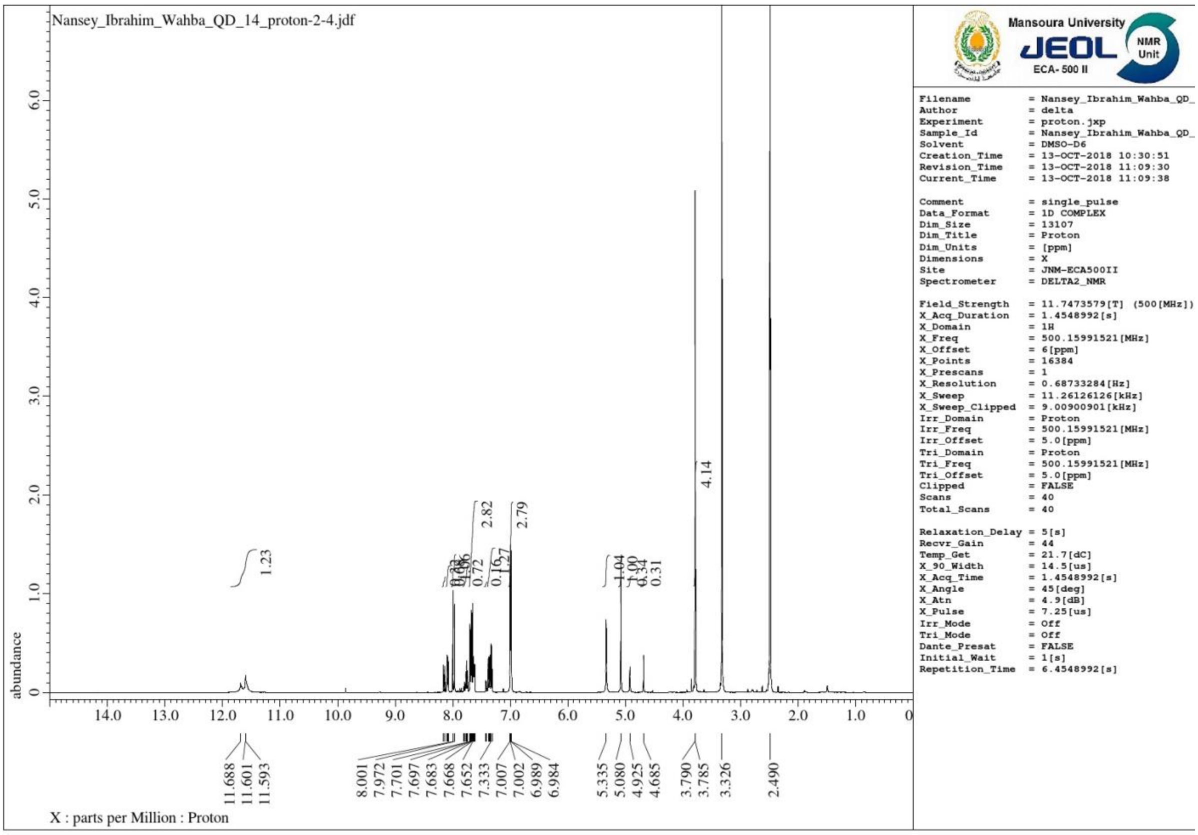

Comp.11a

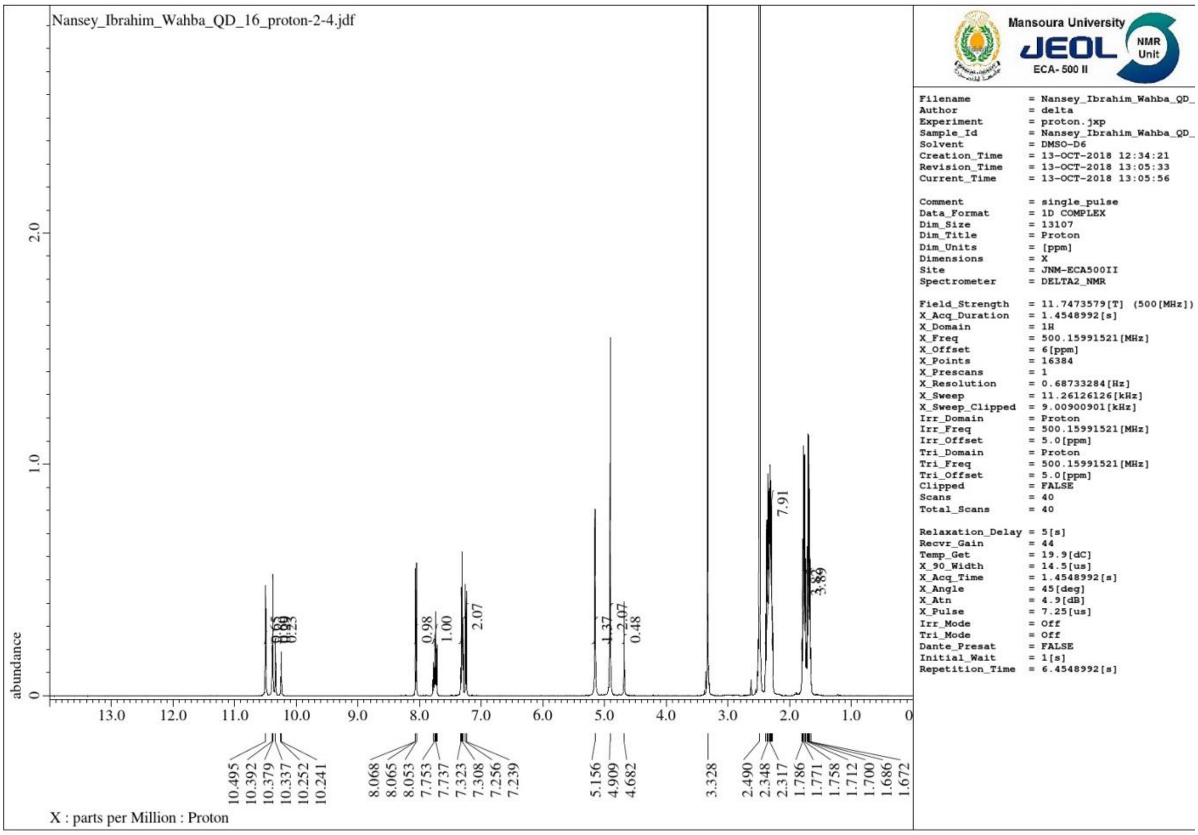
Nansey_Ibrahim_Wahba_QD_16_proton-2-4.jdf
Mansoura University
JEOL
NMR Unit
ECA- 500 II
Filename = Nansey_Ibrahim_Wahba_QD_
Author = delta
Experiment = proton.jxp
Sample_Id = Nansey_Ibrahim_Wahba_QD_
Solvent = DMSO-D6
Creation_Time = 13-OCT-2018 12:34:21
Revision_Time = 13-OCT-2018 13:05:33
Current_Time = 13-OCT-2018 13:05:56
Comment = single_pulse
Data_Format = 1D COMPLEX
Dim_Size = 13107
Dim_Title = Proton
Dim_Units = [ppm]
Dimensions = X
Site = JNM-ECA500II
Spectrometer = DELTA2_NMR
Field_Strength = 11.7473579[T] (500[MHz])
X_Acq_Duration = 1.4548992[s]
X_Domain = 1H
X_Freq = 500.15991521[MHz]
X_Offset = 6[ppm]
X_Points = 16384
X_Prescans = 1
X_Resolution = 0.68733284[Hz]
X_Sweep = 11.26126126[kHz]
X_Sweep_Clipped = 9.00900901[kHz]
Irr_Domain = Proton
Irr_Freq = 500.15991521[MHz]
Irr_Offset = 5.0[ppm]
Tri_Domain = Proton
Tri_Freq = 500.15991521[MHz]
Tri_Offset = 5.0[ppm]
Clipped = FALSE
Scans = 40
Total_Scans = 40
Relaxation_Delay = 5[s]
Recvr_Gain = 44
Temp_Get = 19.9[dC]
X_90_Width = 14.5[us]
X_Acq_Time = 1.4548992[s]
X_Angle = 45[deg]
X_Atn = 4.9[dB]
X_Pulse = 7.25[us]
Irr_Mode = Off
Tri_Mode = Off
Dante_Presat = FALSE
Initial_Wait = 1[s]
Repetition_Time = 6.4548992[s]
abundance
0
1.0
2.0
13.0 12.0 11.0 10.0 9.0 8.0 7.0 6.0 5.0 4.0 3.0 2.0 1.0 0
0.98
1.00
2.07
1.37
2.07
0.48
7.91
3.89
0.23
10.495 10.392 10.379 10.337 10.252 10.241
8.068 8.065 8.053 7.753 7.737 7.323 7.308 7.256 7.239
5.156 4.909 4.682
3.328
2.490 2.348 2.317 1.786 1.771 1.758 1.712 1.700 1.686 1.672
X : parts per Million : Proton

Comp.11b

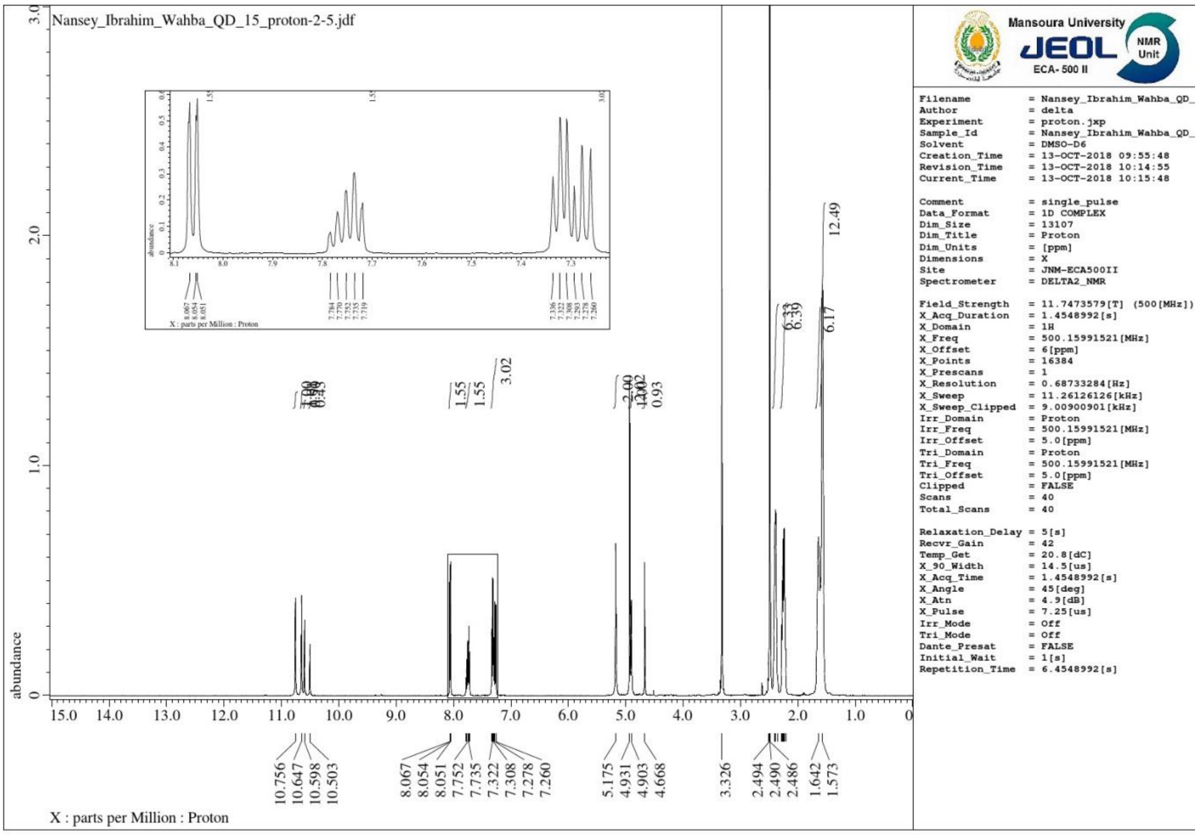

Nansey_Ibrahim_Wahba_QD_15_proton-2-5.jdf
Mansoura University
JEOL
NMR Unit
ECA-500 II
Filename = Nansey_Ibrahim_Wahba_QD_
Author = delta
Experiment = proton.jxp
Sample_Id = Nansey_Ibrahim_Wahba_QD_
Solvent = DMSO-D6
Creation_Time = 13-OCT-2018 09:55:48
Revision_Time = 13-OCT-2018 10:14:55
Current_Time = 13-OCT-2018 10:15:48
Comment = single_pulse
Data_Format = 1D COMPLEX
Dim_Size = 13107
Dim_Title = Proton
Dim_Units = [ppm]
Dimensions = X
Site = JNM-ECA500II
Spectrometer = DELTA2_NMR
Field_Strength = 11.7473579[T] (500[MHz])
X_Acq_Duration = 1.4548992[s]
X_Domain = 1H
X_Freq = 500.15991521[MHz]
X_Offset = 6[ppm]
X_Points = 16384
X_Prescans = 1
X_Resolution = 0.68733284[Hz]
X_Sweep = 11.26126126[kHz]
X_Sweep_Clipped = 9.00900901[kHz]
Irr_Domain = Proton
Irr_Freq = 500.15991521[MHz]
Irr_Offset = 5.0[ppm]
Tri_Domain = Proton
Tri_Freq = 500.15991521[MHz]
Tri_Offset = 5.0[ppm]
Clipped = FALSE
Scans = 40
Total_Scans = 40
Relaxation_Delay = 5[s]
Recvr_Gain = 42
Temp_Get = 20.8[dC]
X_90_Width = 14.5[us]
X_Acq_Time = 1.4548992[s]
X_Angle = 45[deg]
X_Atn = 4.9[dB]
X_Pulse = 7.25[us]
Irr_Mode = Off
Tri_Mode = Off
Dante_Presat = FALSE
Initial_Wait = 1[s]
Repetition_Time = 6.4548992[s]
abundance
X : parts per Million : Proton
10.756 10.647 10.598 10.503
8.067 8.054 8.051 7.752 7.735 7.322 7.308 7.278 7.260
5.175 4.931 4.903 4.668
3.326
2.494 2.490 2.486
1.642 1.573
1.55 1.55 3.02
2.00 0.93
6.33 6.39 6.17 12.49

Comp.11c

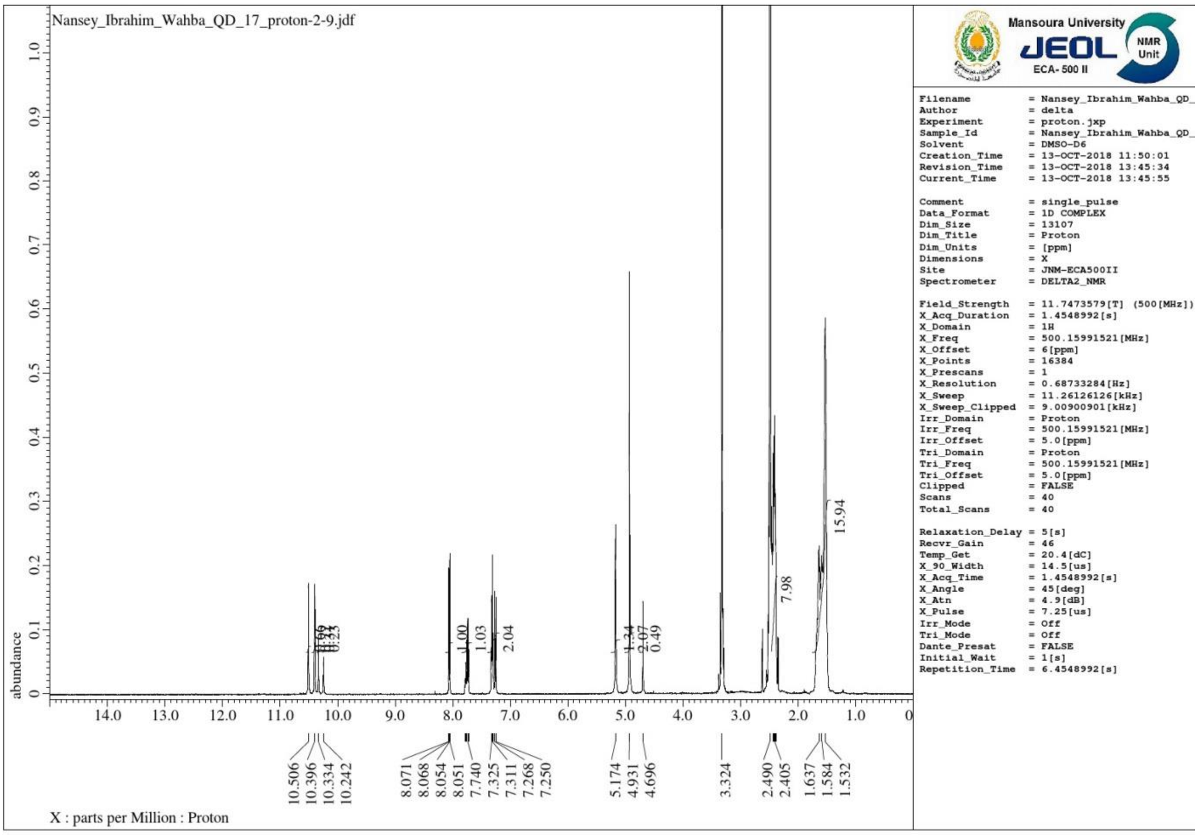
Nansey_Ibrahim_Wahba_QD_17_proton-2-9.jdf
abundance
X : parts per Million : Proton
Mansoura University
JEOL
NMR Unit
ECA- 500 II
Filename = Nansey_Ibrahim_Wahba_QD_
Author = delta
Experiment = proton.jxp
Sample_Id = Nansey_Ibrahim_Wahba_QD_
Solvent = DMSO-D6
Creation_Time = 13-OCT-2018 11:50:01
Revision_Time = 13-OCT-2018 13:45:34
Current_Time = 13-OCT-2018 13:45:55
Comment = single_pulse
Data_Format = 1D COMPLEX
Dim_Size = 13107
Dim_Title = Proton
Dim_Units = [ppm]
Dimensions = X
Site = JNM-ECA500II
Spectrometer = DELTA2_NMR
Field_Strength = 11.7473579[T] (500[MHz])
X_Acq_Duration = 1.4548992[s]
X_Domain = 1H
X_Freq = 500.15991521[MHz]
X_Offset = 6[ppm]
X_Points = 16384
X_Prescans = 1
X_Resolution = 0.68733284[Hz]
X_Sweep = 11.26126126[kHz]
X_Sweep_Clipped = 9.00900901[kHz]
Irr_Domain = Proton
Irr_Freq = 500.15991521[MHz]
Irr_Offset = 5.0[ppm]
Tri_Domain = Proton
Tri_Freq = 500.15991521[MHz]
Tri_Offset = 5.0[ppm]
Clipped = FALSE
Scans = 40
Total_Scans = 40
Relaxation_Delay = 5[s]
Recvr_Gain = 46
Temp_Get = 20.4[dC]
X_90_Width = 14.5[us]
X_Acq_Time = 1.4548992[s]
X_Angle = 45[deg]
X_Atn = 4.9[dB]
X_Pulse = 7.25[us]
Irr_Mode = Off
Tri_Mode = Off
Dante_Presat = FALSE
Initial_Wait = 1[s]
Repetition_Time = 6.4548992[s]
10.506
10.396
10.334
10.242
8.071
8.068
8.054
8.051
7.740
7.325
7.311
7.268
7.250
5.174
4.931
4.696
3.324
2.490
2.405
1.637
1.584
1.532
1.00
1.03
2.04
1.34
2.07
0.49
7.98
15.94

Comp.12a

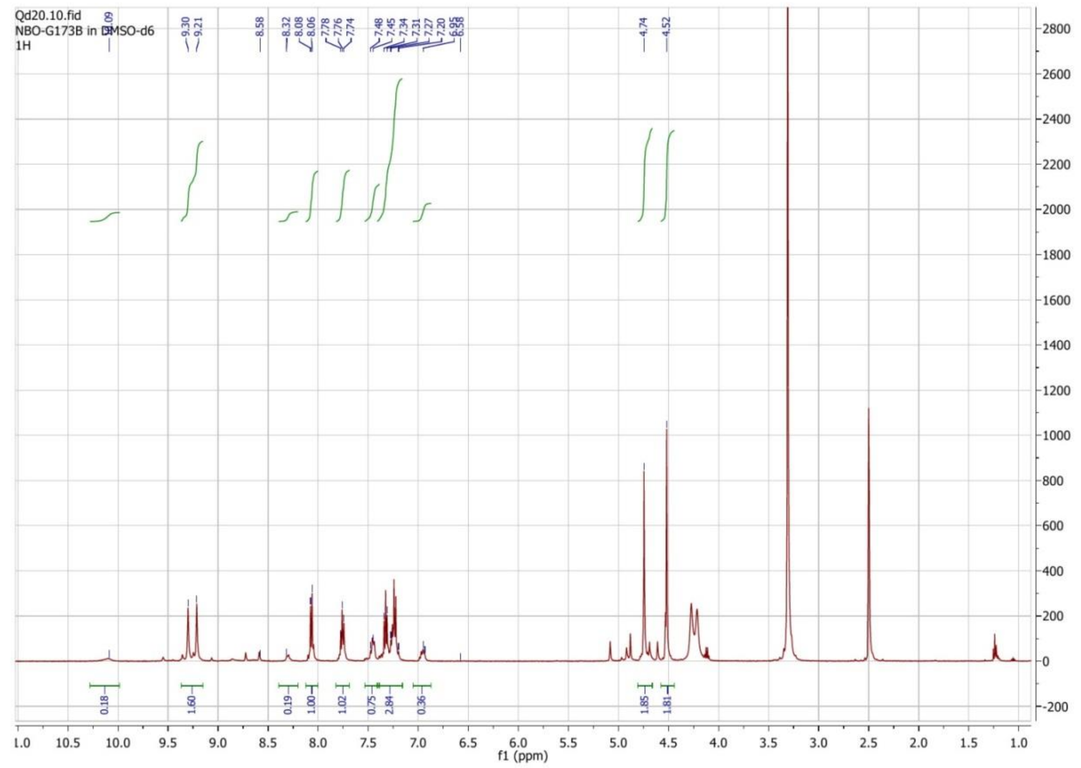

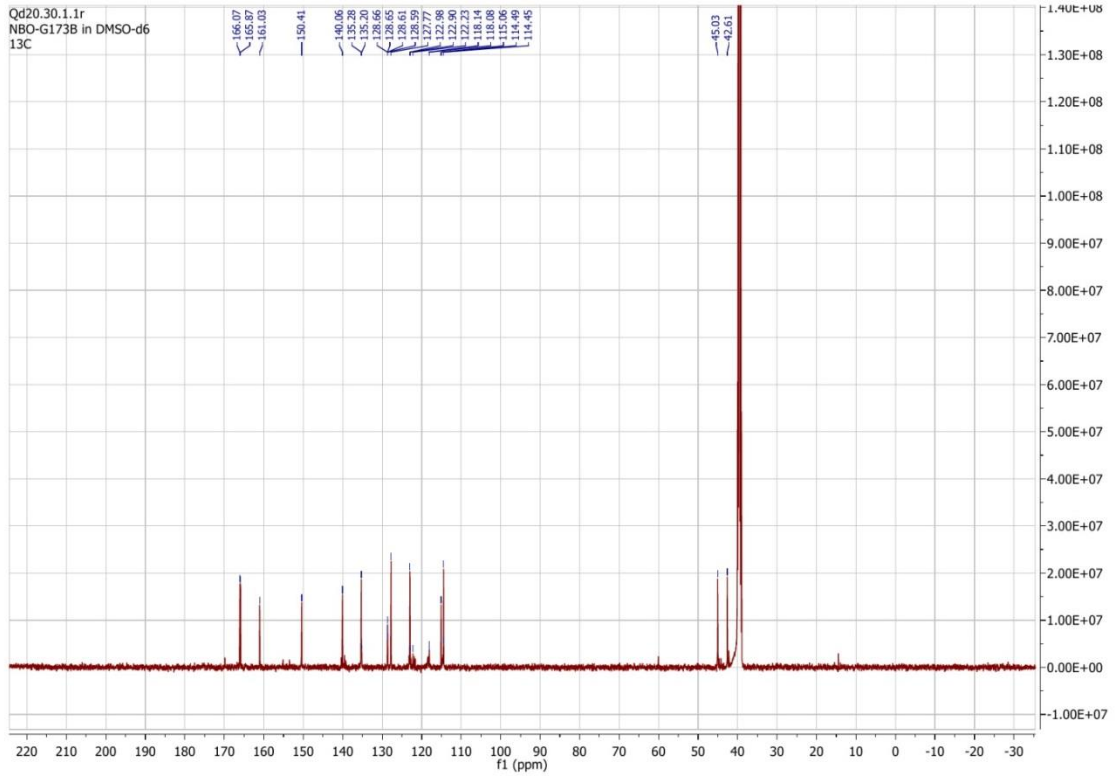

Comp.12b

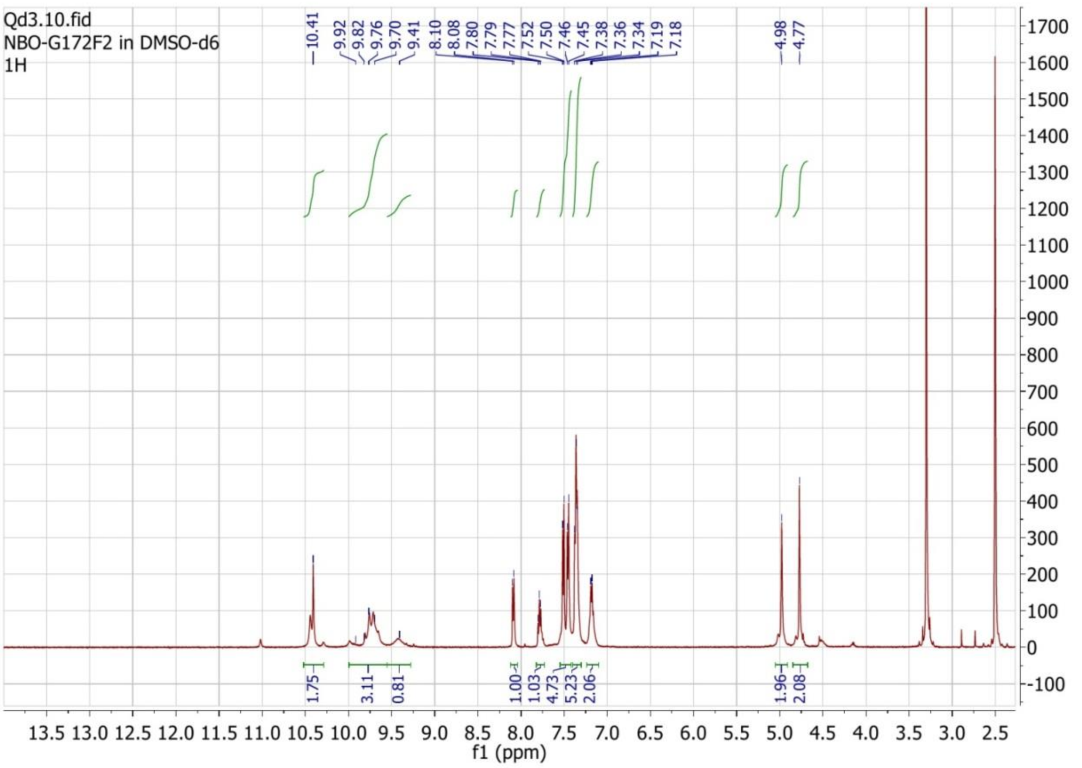
Qd3.10.fid
NBO-G172F2 in DMSO-d6
1H
10.41
9.92
9.82
9.76
9.70
9.41
8.10
8.08
7.80
7.79
7.77
7.52
7.50
7.46
7.45
7.38
7.36
7.34
7.19
7.18
4.98
4.77
1.75
3.11
0.81
1.00
1.03
4.73
5.23
2.06
1.96
2.08
f1 (ppm)

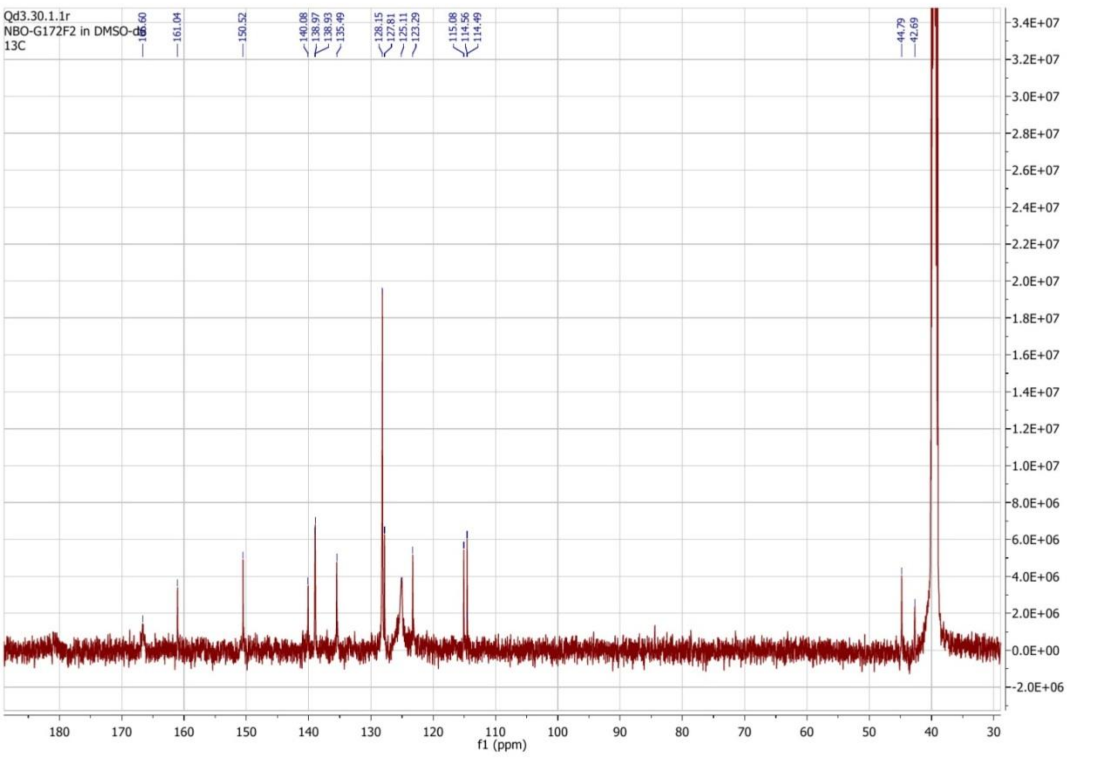
Qd3.30.1.1r
NBO-G172F2 in DMSO-d6
13C
161.04
150.52
140.08
138.97
138.93
135.49
128.15
127.81
125.11
123.29
115.08
114.56
114.49
44.79
42.69
f1 (ppm)

Comp.13

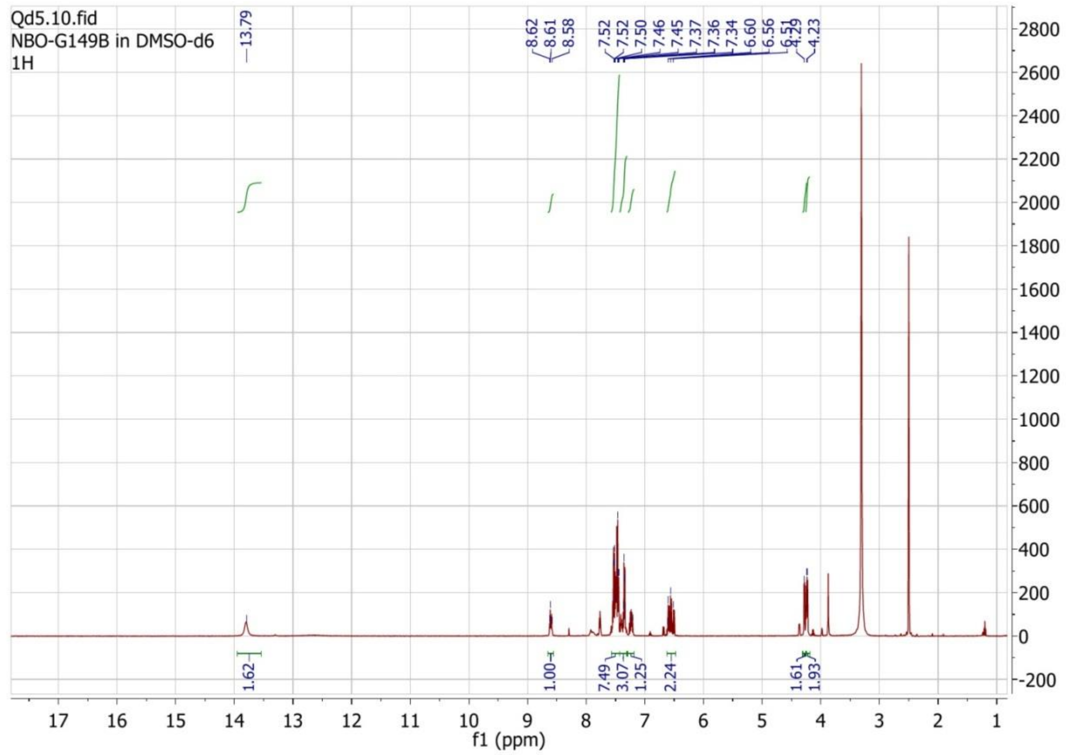
Qd5.10.fid
NBO-G149B in DMSO-d6
1H
13.79
8.62
8.61
8.58
7.52
7.52
7.50
7.46
7.45
7.37
7.36
7.34
6.60
6.56
6.51
4.29
4.23
1.62
1.00
7.49
3.07
1.25
2.24
1.61
1.93
f1 (ppm)

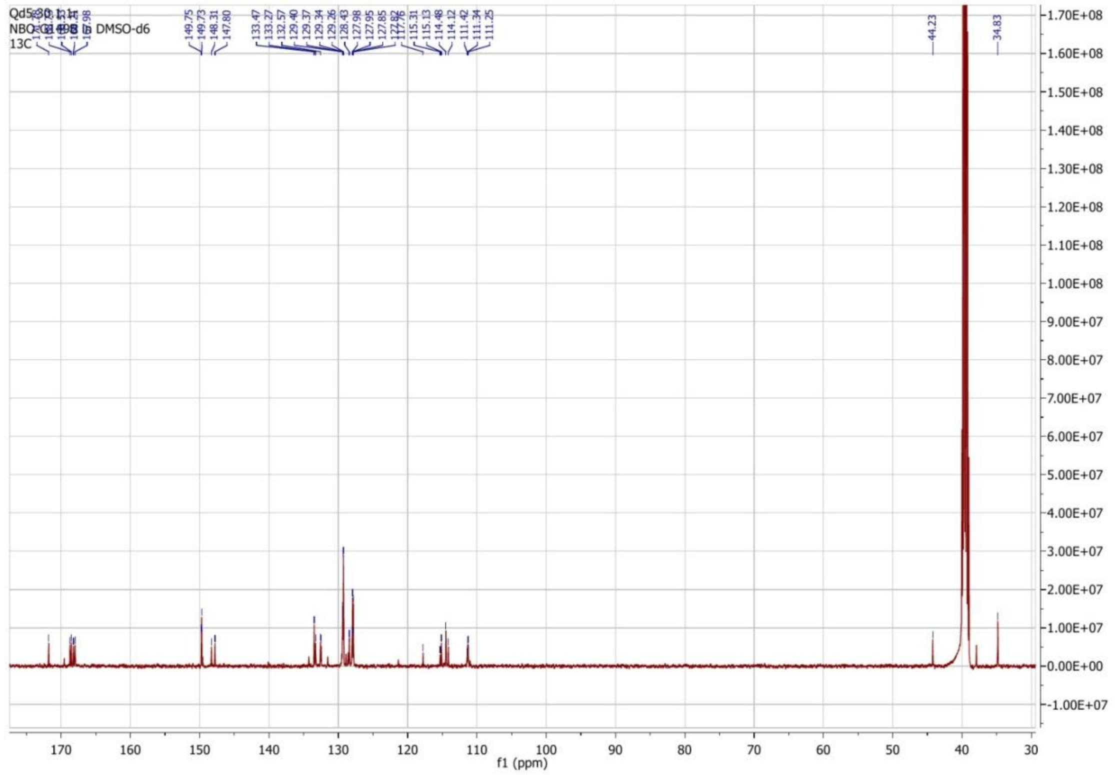
DMSO-d6
13C
149.75
149.73
148.31
147.80
133.47
133.27
132.57
129.40
129.37
129.34
129.26
128.43
127.98
127.95
127.85
115.31
115.13
114.48
114.12
111.42
111.34
111.25
44.23
34.83
f1 (ppm)

Comp.14a

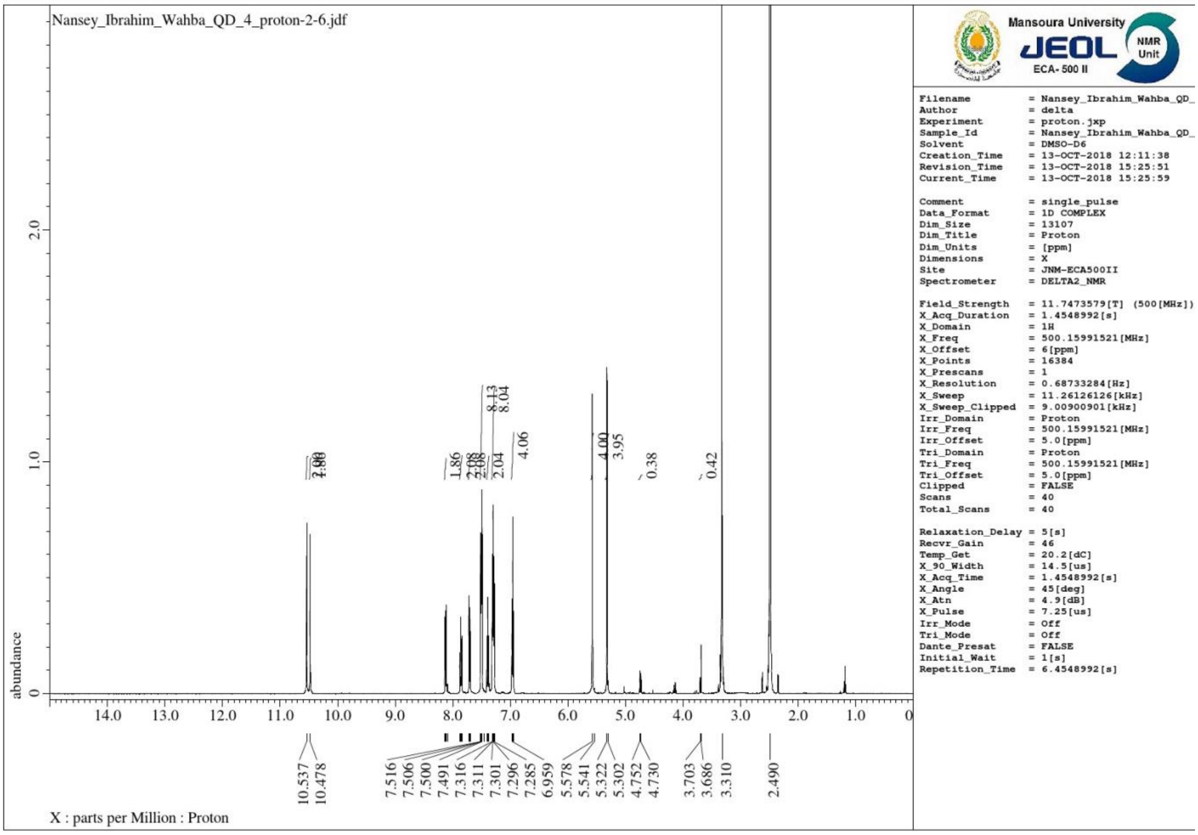

Nansey_Ibrahim_Wahba_QD_4_proton-2-6.jdf
Mansoura University
JEOL
NMR Unit
ECA- 500 II
Filename = Nansey_Ibrahim_Wahba_QD_
Author = delta
Experiment = proton.jxp
Sample_Id = Nansey_Ibrahim_Wahba_QD_
Solvent = DMSO-D6
Creation_Time = 13-OCT-2018 12:11:38
Revision_Time = 13-OCT-2018 15:25:51
Current_Time = 13-OCT-2018 15:25:59
Comment = single_pulse
Data_Format = 1D COMPLEX
Dim_Size = 13107
Dim_Title = Proton
Dim_Units = [ppm]
Dimensions = X
Site = JNM-ECA500II
Spectrometer = DELTA2_NMR
Field_Strength = 11.7473579[T] (500[MHz])
X_Acq_Duration = 1.4548992[s]
X_Domain = 1H
X_Freq = 500.15991521[MHz]
X_Offset = 6[ppm]
X_Points = 16384
X_Prescans = 1
X_Resolution = 0.68733284[Hz]
X_Sweep = 11.26126126[kHz]
X_Sweep_Clipped = 9.00900901[kHz]
Irr_Domain = Proton
Irr_Freq = 500.15991521[MHz]
Irr_Offset = 5.0[ppm]
Tri_Domain = Proton
Tri_Freq = 500.15991521[MHz]
Tri_Offset = 5.0[ppm]
Clipped = FALSE
Scans = 40
Total_Scans = 40
Relaxation_Delay = 5[s]
Recvr_Gain = 46
Temp_Get = 20.2[dC]
X_90_Width = 14.5[us]
X_Acq_Time = 1.4548992[s]
X_Angle = 45[deg]
X_Atn = 4.9[dB]
X_Pulse = 7.25[us]
Irr_Mode = Off
Tri_Mode = Off
Dante_Presat = FALSE
Initial_Wait = 1[s]
Repetition_Time = 6.4548992[s]
abundance
X : parts per Million : Proton
10.537 10.478 7.516 7.506 7.500 7.491 7.316 7.311 7.301 7.296 7.285 6.959 5.578 5.541 5.322 5.302 4.752 4.730 3.703 3.686 3.310 2.490
1.86 8.13 8.04 2.04 4.06 4.00 3.95 0.38 0.42

Comp.14b

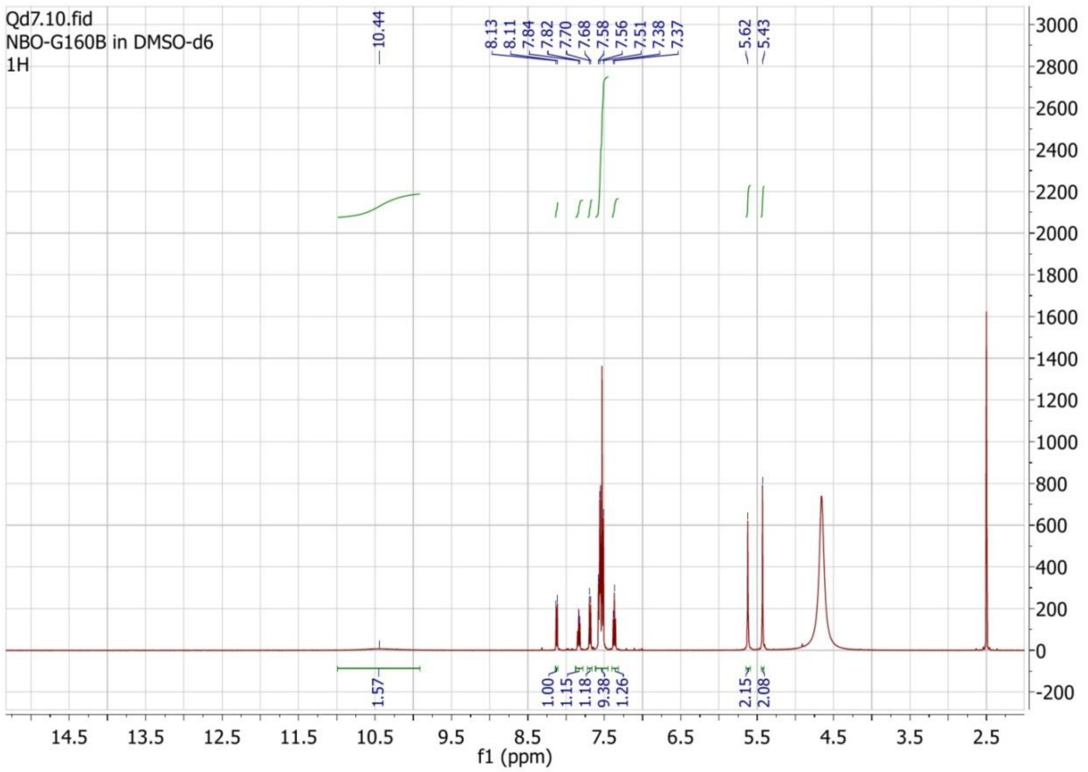
Qd7.10.fid
NBO-G160B in DMSO-d6
1H
10.44
8.13
8.11
7.84
7.82
7.70
7.68
7.58
7.56
7.51
7.38
7.37
5.62
5.43
1.57
1.00
1.15
1.18
9.38
1.26
2.15
2.08
f1 (ppm)

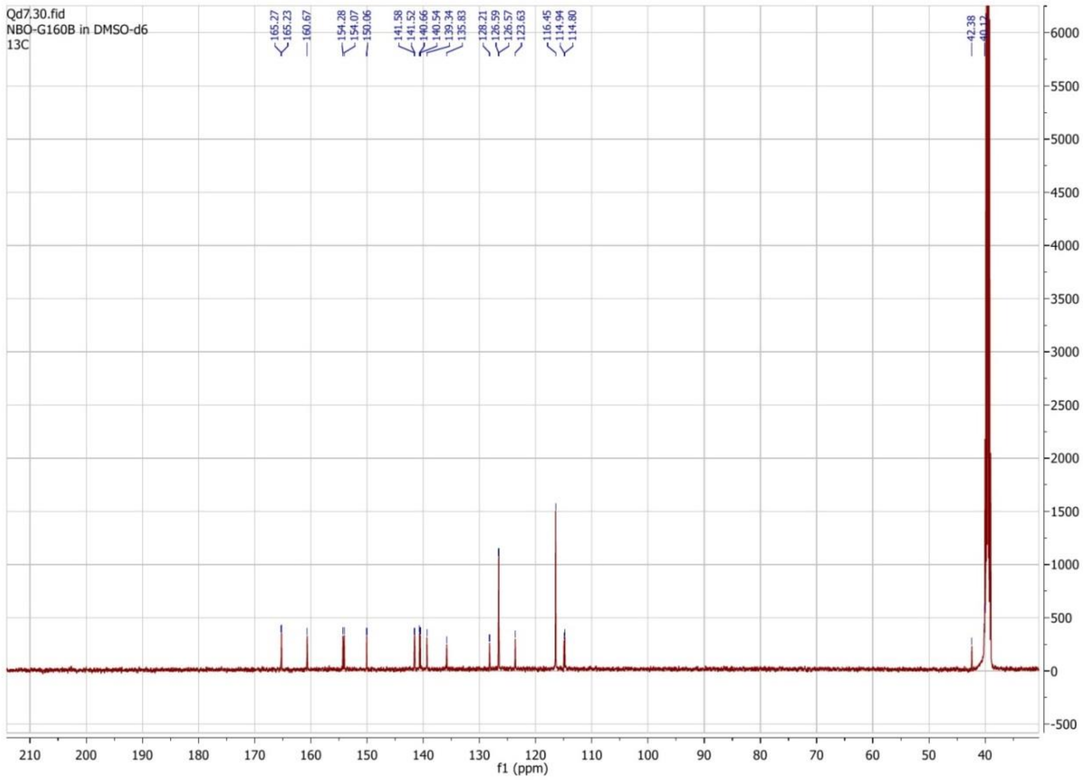
Qd7.30.fid
NBO-G160B in DMSO-d6
13C
165.27
165.23
160.67
154.28
154.07
150.06
141.58
141.52
140.66
140.54
139.34
135.83
128.21
126.59
126.57
123.63
116.45
114.94
114.80
42.38
40.12
f1 (ppm)

Comp.15

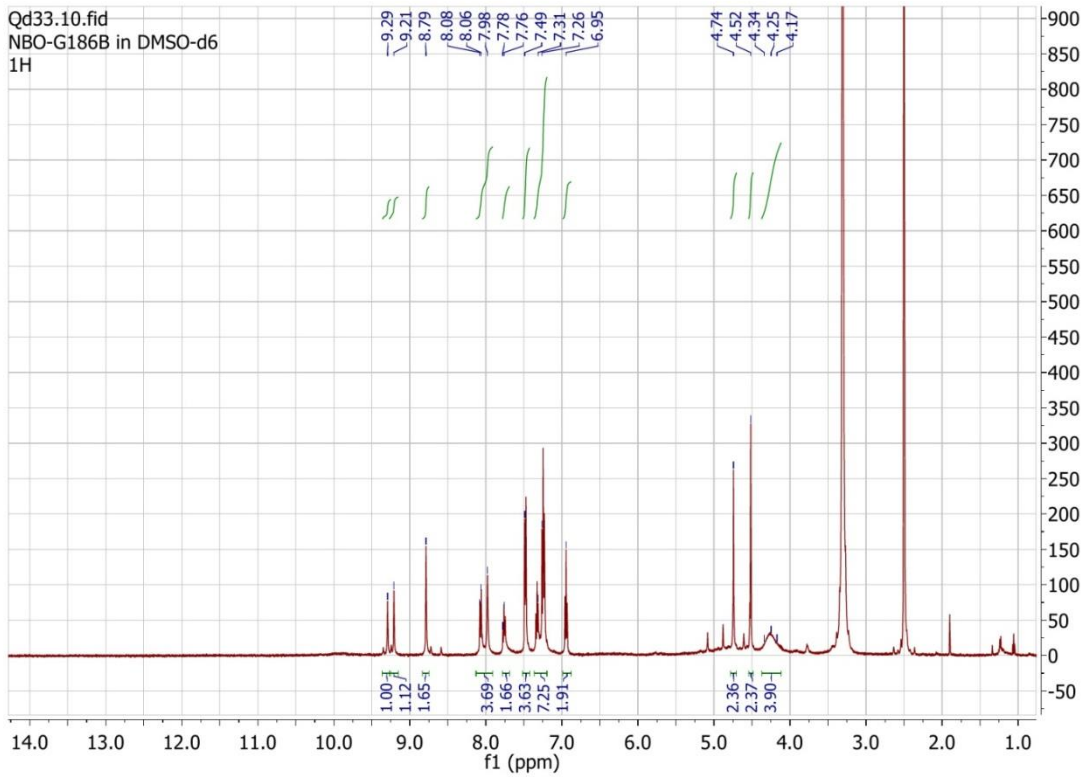
Qd33.10.fid
NBO-G186B in DMSO-d6
1H
9.29
9.21
8.79
8.08
8.06
7.98
7.78
7.76
7.49
7.31
7.26
6.95
4.74
4.52
4.34
4.25
4.17
1.00
1.12
1.65
3.69
1.66
3.63
7.25
1.91
2.36
2.37
3.90
f1 (ppm)

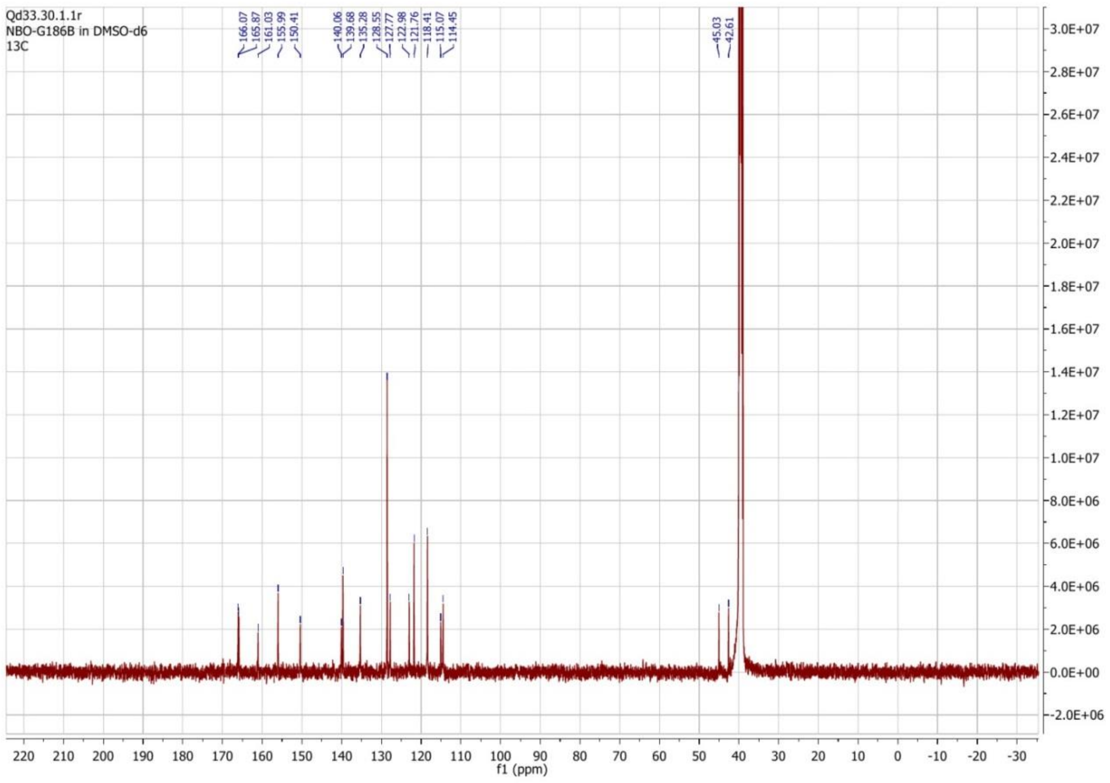
Qd33.30.1.1r
NBO-G186B in DMSO-d6
13C
166.07
165.87
161.03
155.99
150.41
140.06
139.68
135.28
128.55
127.77
122.98
121.76
118.41
115.07
114.45
45.03
42.61
f1 (ppm)

Comp.16

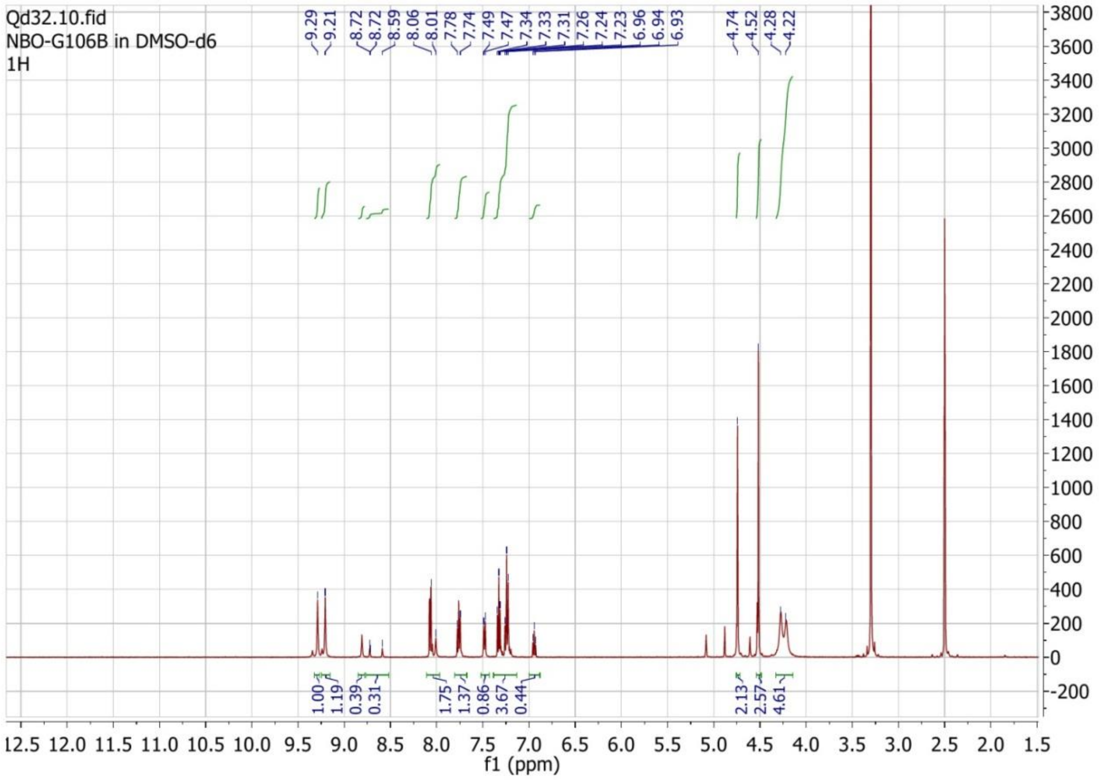
Qd32.10.fid
NBO-G106B in DMSO-d6
1H
9.29
9.21
8.72
8.72
8.59
8.06
8.01
7.78
7.74
7.49
7.47
7.34
7.33
7.31
7.26
7.24
7.23
6.96
6.94
6.93
4.74
4.52
4.28
4.22
1.00
1.19
0.39
0.31
1.75
1.37
0.86
3.67
0.44
2.13
2.57
4.61
f1 (ppm)

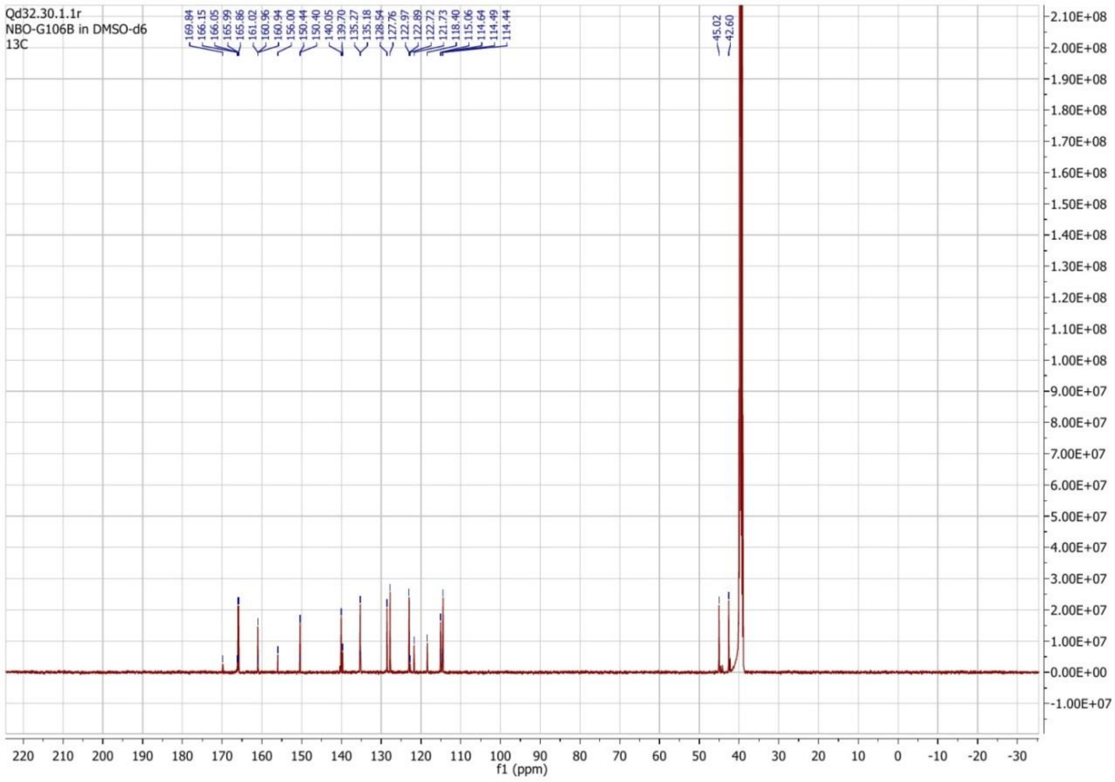
Qd32.30.1.1r
NBO-G106B in DMSO-d6
13C
169.84
166.15
166.05
165.99
165.86
161.02
160.96
160.94
156.00
150.44
150.40
140.05
139.70
135.27
135.18
128.54
127.76
122.97
122.89
122.72
121.73
118.40
115.06
114.64
114.49
114.44
45.02
42.60
f1 (ppm)
